# On the optimal certification of von Neumann measurements

**DOI:** 10.1038/s41598-021-81325-1

**Published:** 2021-02-11

**Authors:** Paulina Lewandowska, Aleksandra Krawiec, Ryszard Kukulski, Łukasz Pawela, Zbigniew Puchała

**Affiliations:** 1 Institute of Theoretical and Applied Informatics, Polish Academy of Sciences, ul. Bałtycka 5, 44-100 Gliwice, Poland; 2 Faculty of Physics, Astronomy and Applied Computer Science, Jagiellonian University, ul. Łojasiewicza 11, 30-348 Kraków, Poland

**Keywords:** Quantum information, Quantum mechanics, Qubits

## Abstract

In this report we study certification of quantum measurements, which can be viewed as the extension of quantum hypotheses testing. This extension involves also the study of the input state and the measurement procedure. Here, we will be interested in two-point (binary) certification scheme in which the null and alternative hypotheses are single element sets. Our goal is to minimize the probability of the type II error given some fixed statistical significance. In this report, we begin with studying the two-point certification of pure quantum states and unitary channels to later use them to prove our main result, which is the certification of von Neumann measurements in single-shot and parallel scenarios. From our main result follow the conditions when two pure states, unitary operations and von Neumann measurements cannot be distinguished perfectly but still can be certified with a given statistical significance. Moreover, we show the connection between the certification of quantum channels or von Neumann measurements and the notion of *q*-numerical range.

## Introduction

The validation of sources producing quantum states and measurement devices, which are involved in quantum computation workflows, is a necessary step of quantum technology^1–3^. The search for practical and reliable tools for validation of quantum architecture has attracted a lot of attention in recent years^4–8^. Rapid technology development and increasing interest in quantum computers paved the way towards creating more and more efficient validation methods of Noisy Intermediate-Scale Quantum devices (NISQ)^9,10^. Such a growth comes along with ever-increasing requirements for the precision of the components of quantum devices. The tasks of ensuring the correctness of quantum devices are referred to as *validation*.

Let us begin with sketching the problem of validation of quantum architectures. Imagine you are given a black box and are promised two things. First, it contains a pure quantum state (or a unitary matrix or a von Neumann POVM), and second, it contains one of two possible choices of these objects. The owner of the box, Eve, tells you which of the two possibilities is contained within the box. Yet, for some reason, you do not completely trust her and decide to perform some kind of hypothesis testing scheme on the black box. You decide to take Eve’s promise as the null hypothesis, $H_0$, for this scheme and the second of the possibilities as the alternative hypothesis, $H_1$. Since now you own the box and are free to proceed as you want, you need to prepare some input into the box and perform a measurement on the output. A particular input state and final measurement (or only the measurement, for the case when the box contains a quantum state) will be called a certification strategy. Of course, just like in classical hypothesis testing, in our certification scheme we have two possible types of errors. The type I error happens if we reject the null hypothesis when it was in reality true. The type II error happens if we accept the null hypothesis when we should have rejected it. The main aim of certification is finding the optimal strategy which minimizes one type of error when the other one is fixed. In this work we are interested in the minimization of the type II error given a fixed type I error. This approach will be called *certification*.

Certification of quantum objects is closely related with the other well-know method of validation, that is the problem of discrimination of those objects. Intuitively, in the discrimination problem we are given one of two quantum objects sampled according to a given a priori probability distribution. Hence, the probability of making an error in the discrimination task is equal to the average of the type I and type II errors over the assumed probability distribution. Therefore, the discrimination problem can be seen as *symmetric distinguishability*, as opposed to certification, that is *asymmetric distinguishability*. In other words, the main difference between both approaches is that the main task of discrimination is the minimization over the average of both types of possible errors while the certification concerns the minimization over one type of error when the bound of the other one is assumed.

While in the basic version of the aforementioned scenario we focus on the case when the validated quantum object can be used exactly once, one can consider also the situation in which this object can be utilized multiple times in various configurations. In the parallel scheme the validated object can be used many times, but no processing can be performed between the usages of this object. In the adaptive scenario however, we are allowed to perform any processing we want between the uses of the validated quantum object.

The problem of discrimination of quantum states and channels was solved analytically by Helstrom a few decades ago in^11,12^. The multiple-shot scenario of the discrimination of quantum states was studied in^13,14^ whereas the discrimination of quantum channels in the multiple-shot scenario was investigated for example in^15–19^. Examples of channels which cannot be discriminated perfectly in the parallel scheme, but nonetheless can be discriminated perfectly using the adaptive approach, were discussed in^20,21^. The work^22^ paved the way for studying the discrimination of quantum measurements. Therefore, this work can be seen as a natural extension of our works^23,24^, where we studied the discrimination of von Neumann measurements in single and multiple-shot scenarios, respectively. Nevertheless, one can also consider a scenario in which we are allowed to obtain an inconclusive answer. Therefore, we arrive at the unambiguous discrimination of quantum operations discussed in^24,25^.

The problem of certification of quantum states and channels has not been studied as exhaustively as their discrimination. The certification of quantum objects was first studied by Helstrom in^11^, where the problem of pure state certification was considered. Further, certification schemes were established to the case of mixed states in^26^. A natural extension of quantum state certification is the certification of unitary operations. This problem was solved in^27^. Considerations about multiple-shot scenario of certification of quantum states and unitary channels were investigated in^27^.

All the above-mentioned approaches towards the certification were considered in a finite number of steps. Another common approach involves studying certification of quantum objects in the asymptotic regime^28–30^ which assumes that the number of copies of the given quantum object goes to infinity. It focuses on studying the convergence of the probability of making one type of error while a bound on the second one is assumed. This task is strictly related with the term of relative entropy and its asymptotic behavior^31^. For a more general overview of quantum certification we refer the reader to^32,33^.

This work will begin with recalling one-shot certification scenario which will later be extended to the multiple-shot case. For this purpose, we will often make use of the terms of numerical range and *q*-numerical range as essential tools in the proofs^34–37^. More specifically, one of our results presented in this work is a geometric interpretation of the formula for minimized probability of the type II error in the problem of certification of unitary channels, which is strictly connected with the notion of *q*-numerical range. Later, basing on the results on the certification of unitary channels we will extend these considerations to the problem of certification of von Neumann measurements. It will turn out that the formula for minimized probability of the type II error can also be connected with the notion of *q*-numerical range. On top of that, we will show that entanglement can significantly improve the certification of von Neumann measurements. Eventually, we will prove that the parallel certification scheme is optimal.

This work is organized as follows. We begin with preliminaries in  “Preliminaries” section. Then, in  “Two-point certification of pure states” section we present the two-point certification of pure quantum states. Certification of unitary channels is discussed in  “Certification of unitary channels” section. After presenting the known results we introduce geometrical interpretation of the problem of certification of unitary channels, expressed in terms of *q*-numerical range. The certification of von Neumann measurements is studied in  “Two-point certification of von Neumann measurements” section and our main result is stated therein as Theorem 3.  “Parallel multiple-shot certification” section generalizes the results on certification to the multiple-shot scenario and the optimality of the parallel scheme for certification of von Neumann measurements is presented as Theorem 5.

## Preliminaries

Let $M_{d_1,d_2}$ be the set of all matrices of dimension $d_1 \times d_2$ over the field $\mathbb {C}$. For the sake of simplicity, square matrices will be denoted by $M_d$. The set of quantum states, that is positive semidefinite operators having trace equal to one, will be denoted $\mathcal {D}_d$. By default, when we write $|\psi \rangle , |\varphi \rangle$, we mean normalized pure states, unless we mention otherwise. The subset of $M_d$ consisting of unitary matrices will be denoted by $\mathcal {U}_d$, while its subgroup of diagonal unitary operators will be denoted by $\mathcal {D}\mathcal {U}_d$. Let $U \in \mathcal {U}_d$ be a unitary matrix. A unitary channel $\Phi _{U}$ is defined as $\Phi _U(\cdot ) = U \cdot U^\dagger$. A general quantum measurement, that is a positive operator valued measure (POVM) $\mathcal {P}$ is a collection of positive semidefinite operators $\{E_1, \ldots , E_m \}$ called *effects*, which sum up to identity, i.e. $\, \, \sum _{i=1}^m E_i = {\mathbbm{1}}$. If all the effects are rank-one projection operators, then such a measurement is called von Neumann measurement. Every von Neumann measurement can be parameterized by a unitary matrix and hence we will use the notation $\mathcal {P}_{U}$ for a von Neumann measurement with effects $\{|u_1\rangle \langle u_1|, \ldots , |u_d\rangle \langle u_d|\}$, where $|u_i\rangle$ is the *i*-th column of the unitary matrix *U*. The action of quantum measurement $\mathcal {P}_{U}$ on some state $\rho \in {\mathcal {D}}_d$ can be expressed as the action of a quantum channel

1 $$\begin{aligned} \mathcal {P}_{U} : \rho \rightarrow \sum _{i=1}^d \langle u_i| \rho |u_i\rangle |i\rangle \langle i|. \end{aligned}$$

As mentioned in the “Introduction” section, in this work we focus on two-point hypothesis testing of quantum objects. The starting point towards the certification of quantum objects is the hypothesis testing of quantum states. Let $H_0$ be a null hypothesis which states that the obtained state was $|\psi \rangle$, while the alternative hypothesis, $H_1$, states that the obtained state was $|\varphi \rangle$. The certification is performed by the use of a binary measurement $\{ \Omega , {\mathbbm{1}}- \Omega \}$, where the effect $\Omega$ corresponds to accepting the null hypothesis and ${\mathbbm{1}}-\Omega$ accepts the alternative hypothesis. In this work we will be considering only POVMs with two effects of this form. Therefore the effect $\Omega$ uniquely determines the POVM and hence we will be using the words measurement and effect interchangeably.

Assume we have a fixed measurement $\Omega$. We introduce the probability of the type I error, $p_\text {I}(\Omega )$, that is the probability of rejecting the null hypothesis when in fact it was true, as

2 $$\begin{aligned} p_\text {I}(\Omega ) = {{\,\text{tr}\,}}\left( ({\mathbbm{1}}-\Omega ) |\psi \rangle \langle \psi | \right) = 1 - {{\,\text{tr}\,}}\left( \Omega |\psi \rangle \langle \psi | \right) . \end{aligned}$$

The type II error, $p_{\text {II}}(\Omega )$, that is the probability of accepting the null hypothesis $H_0$ when in reality $H_1$ occurred, is defined as

3 $$\begin{aligned} p_\text {II}(\Omega ) = {{\,\text{tr}\,}}\left( \Omega |\varphi \rangle \langle \varphi | \right) . \end{aligned}$$

In the remainder of this work we will assume the statistical significance $\delta \in [0,1]$, that is the probability of the type I error will be upper-bounded by $\delta$. Our goal will be to find the most powerful test, that is to minimize the probability of the type II error by finding the optimal measurement, which we will denote as $\Omega _0$. Such $\Omega _0$, which minimizes $p_\text {II}(\Omega )$ while assuming the statistical significance $\delta$, will be called an *optimal measurement*. The minimized probability of type II error will be denoted by

4 $$\begin{aligned} p_{\text {II}} :=\min _{\Omega : p_{\text {I}}(\Omega ) \le \delta } p_{\text {II}}(\Omega ). \end{aligned}$$

While certifying quantum channels and von Neumann measurements, we will also need to minimize over input states. Let a channel $\Phi _0$ correspond to hypothesis $H_0$ and $\Phi _1$ correspond to hypothesis $H_1$. We define

5 $$\begin{aligned} p_{\text {I}}^{|\psi \rangle }(\Omega )&= {{\,\text{tr}\,}}\left( ({\mathbbm{1}}-\Omega )\Phi _0(|\psi \rangle \langle \psi |) \right), \\ p_{\text {II}}^{|\psi \rangle }(\Omega )&= {{\,\text{tr}\,}}\left( \Omega \Phi _1(|\psi \rangle \langle \psi |) \right) . \end{aligned}$$

Naturally, for each input state we can consider minimized probability of type II error, that is

6 $$\begin{aligned} p_{\text {II}}^{|\psi \rangle }=\min _{\Omega : p_{\text {I}}^{|\psi \rangle }(\Omega ) \le \delta } p_{\text {II}}^{|\psi \rangle }(\Omega ). \end{aligned}$$

Finally, we will be interested in calculating optimized probability of type II error over all input states. This will be denoted as

7 $$\begin{aligned} p_{\text {II}} :=\min _{|\psi \rangle } p_{\text {II}}^{|\psi \rangle }. \end{aligned}$$

Note that the symbol $p_{\text {II}}$ is used in two contexts. In the problem of certification of states the minimization is performed only over measurements $\Omega$, while in the problem of certification of unitary channels and von Neumann measurements the minimization is over both measurements $\Omega$ and input states $|\psi \rangle$. In other words, $p_{\text {II}}$ is equal to the optimized probability of the type II error in certain certification problem.

The input state which minimizes $p_{\text {II}}$ will be called an *optimal state*. We will use the term *optimal strategy* to denote both the optimal state and the optimal measurement.

Now, we introduce a basic toolbox for studying the certification of quantum objects which is strictly related with the problem of discrimination of quantum channels. First, we will be using the notion of the diamond norm. The diamond norm of a superoperator $\Psi$ is defined as

8 $$\begin{aligned} \Vert \Psi \Vert _\diamond :=\max _{\Vert X\Vert _1 = 1} \Vert \left( \Psi \otimes {\mathbbm{1}}\right) (X) \Vert _1. \end{aligned}$$

The celebrated theorem of Helstrom^11^ gives a lower bound on the probability of making an error in distinction in the scenario of symmetric discrimination of quantum channels. The probability of incorrect symmetric discrimination between quantum channels $\Phi$ and $\Psi$ is bounded as follows

9 $$\begin{aligned} p_{e} \ge \frac{1}{2} - \frac{1}{4} \Vert \Phi - \Psi \Vert _\diamond . \end{aligned}$$

Moreover, our results will often make use of the terms of numerical range and *q*-numerical range^37^. The numerical range is a subset of complex plane defined for a matrix $X \in M_d$ as

10 $$\begin{aligned} W(X) := \{ \langle \psi | X |\psi \rangle : \langle {\psi }|{\psi }\rangle =1 \} \end{aligned}$$

while the *q*-numerical range^34–36^ is defined for a matrix $X\in M_d$ as

11 $$\begin{aligned} W_q(X) := \{ \langle \psi | X |\varphi \rangle : \langle {\psi }|{\psi }\rangle = \langle {\varphi }|{\varphi }\rangle = 1, \, \langle {\psi }|{\varphi }\rangle =q,\, q \in \mathbb {C}\}. \end{aligned}$$

The standard numerical range is the special case of *q*-numerical range for $q=1$, that is $W(X) = W_1(X)$. We will use the notation

12 $$\begin{aligned} \nu _{q}(X):= \min \{ |x|: x \in W_q(X) \} \end{aligned}$$

to denote the distance on a complex plane from *q*-numerical range to zero. In the case when $q=1$, we will simply write $\nu (X)$. The main properties of *q*-numerical range are its convexity and compactness^36^. The detailed shape of *q*-numerical range is described in^35^. The properties of *q*-numerical range^15^ that will be used throughout this paper are

13 $$\begin{aligned} W_{q'} \subseteq \frac{q'}{q} W_{q} \quad \text {for} \quad q \le q', \quad q,q' \in \mathbb {R},\end{aligned}$$

and

14 $$\begin{aligned} W_q (X \otimes {\mathbbm{1}}) = W_q(X), \quad q\in \mathbb {R}. \end{aligned}$$

From the above it is easy to see that

15 $$\begin{aligned} \nu _q (X \otimes {\mathbbm{1}}) = \nu _q(X), \quad q\in \mathbb {R}. \end{aligned}$$

In the Supplementary Materials we provide an animation of *q*-numerical range of unitary matrix $U \in \mathcal {U}_3$ with eigenvalues $1, \text {e}^{ \frac{\pi \text {i}}{3}}$ and $\text {e}^{ \frac{2\pi \text {i}}{3}}$ for all parameters $q \in [0,1]$.

## Two-point certification of pure states

In this section we recall the results concerning the certification of pure quantum states. We state the optimized probability of the type II error for the quantum hypothesis testing problem as well as the form of the optimal measurement which should be used for the certification. Although these results may seem quite technical, they will lay the groundwork for studying the certification of unitary channels and von Neumann measurements in further sections.

### Certification scheme

Assume we are given one of two known quantum states either $|\psi \rangle$ or $|\varphi \rangle$. The hypothesis $H_0$ corresponds to the state $|\psi \rangle$, while the alternative hypothesis $H_1$ corresponds to the state $|\varphi \rangle$. In other words, our goal is to decide whether the given state was $|\psi \rangle$ or $|\varphi \rangle$. To make a decision, we need to measure the given state and we are allowed to use any POVM. We will use a quantum measurement with effects $\{\Omega , {\mathbbm{1}}- \Omega \}$, where the first effect $\Omega$ accepts the hypothesis $H_0$ and the second effect ${\mathbbm{1}}-\Omega$ accepts $H_1$. Hence, the probability of obtaining the type I error is given by

16 $$\begin{aligned} p_\text {I}(\Omega ) = \langle \psi | ({\mathbbm{1}}-\Omega ) |\psi \rangle . \end{aligned}$$

The probability of obtaining the type II error to be minimized yields

17 $$\begin{aligned} p_\text {II}=\min _{\Omega : p_{\text {I}}(\Omega ) \le \delta } \langle \varphi | \Omega |\varphi \rangle =:\langle \varphi | \Omega _0 |\varphi \rangle , \end{aligned}$$

where the minimization is performed by finding the optimal measurement $\Omega _0$.

This problem was explored in^11^. However, to keep this work self-consistent we present in Online Appendix A in the Supplementary Materials an alternative version of the proof.

#### Theorem 1.

*Consider the problem of two-point certification of pure quantum states with hypotheses given by*

18 $$\begin{array}{*{20}l} {H_{0} :\;|\psi \rangle ,} \\ {H_{1} :\;|\varphi \rangle .} \\ \end{array}$$

*and statistical significance* $\delta \in [0,1]$. *Then, for the most powerful test, the probability of the type II error* (17) *yields*

19 $$\begin{aligned} p_\text {II} = \left\{ \begin{array}{ll} 0 &{} \text {if} \,\,\,|\langle {\psi }|{\varphi }\rangle | \le \sqrt{\delta }, \\ \left( |\langle {\psi }|{\varphi }\rangle | \sqrt{1-\delta } - \sqrt{1-|\langle {\psi }|{\varphi }\rangle |^2} \sqrt{\delta }\right) ^2 &{} \text {if} \,\,\, |\langle {\psi }|{\varphi }\rangle | > \sqrt{\delta }. \end{array} \right. \end{aligned}$$

The proof of the above theorem is presented in Online Appendix A in the Supplementary Materials. This proof gives a construction of the optimal measurement which minimizes the probability of the type II error. The exact form of such an optimal measurement is stated as the following corollary.

#### Corollary 1.

*The optimal strategy for two-point certification of pure quantum states* $|\psi \rangle$ *and* $|\varphi \rangle$, *with statistical significance* $\delta$ *yields*

1. *if* $|\langle {\psi }|{\varphi }\rangle | \le \sqrt{\delta }$, *then the optimal measurement is given by* $\Omega _0= |\omega \rangle \langle \omega |$, *where* $|\omega \rangle = \frac{|\widetilde{\omega }\rangle }{|||\widetilde{\omega }\rangle ||}$, $|\widetilde{\omega }\rangle = |\psi \rangle - \langle {\varphi }|{\psi }\rangle |\varphi \rangle$;
2. *if* $|\langle {\psi }|{\varphi }\rangle | > \sqrt{\delta }$, *then the optimal measurement is given by* $\Omega _0 = |\omega \rangle \langle \omega |$ *for* $|\omega \rangle = \sqrt{1-\delta } |\psi \rangle - \sqrt{\delta } | \psi ^\perp \rangle$, $|\psi ^\perp \rangle = \frac{|\widetilde{\psi ^\perp }\rangle }{|| |\widetilde{\psi ^\perp } \rangle ||}$, *where* $|\widetilde{\psi ^\perp }\rangle = |\varphi \rangle - \langle {\psi }|{\varphi }\rangle |\psi \rangle$.

## Certification of unitary channels

In this section we will be interested in certification of two unitary channels $\Phi _{U}$ and $\Phi _{V}$ for $U,V \in \mathcal {U}_d$. Without loss of generality we can assume that one of these unitary matrices is the identity matrix and then our task reduces to certification of channels $\Phi _{\mathbbm{1}}$ and $\Phi _U$. In the most general case, we are allowed to use entanglement by adding an additional system. Hence, the null hypothesis $H_0$ yields that the unknown channel is $\Phi _{\mathbbm{1}} \otimes {\mathbbm{1}}$ and the alternative hypothesis $H_1$ yields that the unknown channel is $\Phi _U \otimes {\mathbbm{1}}$.

### Certification scheme

The idea behind the scheme of certification of unitary channels is to reduce this problem to certification of quantum states discussed in the previous section. We prepare some (possibly entangled) input state $|\psi \rangle$ and perform the unknown channel on it. The resulting state is either $\left( {\mathbbm{1}} \otimes {\mathbbm{1}} \right) |\psi \rangle$ or $\left( U \otimes {\mathbbm{1}} \right) |\psi \rangle$. Then, we perform the measurement $\{\Omega , {\mathbbm{1}}- \Omega \}$ and make a decision whether the given channel was $\Phi _{\mathbbm{1}} \otimes {\mathbbm{1}}$ or $\Phi _U \otimes {\mathbbm{1}}$. The effect $\Omega$ corresponds to accepting $H_0$ hypothesis while ${\mathbbm{1}}-\Omega$ corresponds to the alternative hypothesis $H_1$.

The results of minimization of the probability of the type II error over input states $|\psi \rangle$ and measurements $\Omega$ are summarized as the following theorem. This reasoning is based on the results from Theorem 1, while a related study of this problem can be found in^27^.

#### Theorem 2.

*Consider the problem of two-point certification of unitary channels* with hypotheses

20 $$\begin{aligned} &H_0: \ \Phi _{\mathbbm{1}} \otimes {\mathbbm{1}}, \\&H_1: \ \Phi _U \otimes {\mathbbm{1}}. \end{aligned}$$

*and statistical significance* $\delta \in [0,1]$. *Then, for the most powerful test, the probability of the type II error yields*

21 $$\begin{aligned} p_{\text {II}} = \left\{ \begin{array}{ll} 0 &{}\text {if} \,\,\, |\langle \psi _0|U |\psi _0\rangle | \le \sqrt{\delta }, \\ \left( |\langle \psi _0|U |\psi _0\rangle | \sqrt{1-\delta } - \sqrt{1-|\langle \psi _0|U |\psi _0\rangle |^2} \sqrt{\delta }\right) ^2 &{} \text {if} \,\,\, |\langle \psi _0|U |\psi _0\rangle |> \sqrt{\delta }, \end{array} \right. \end{aligned}$$

*where* $$|\psi _0\rangle \in \arg \min _{|\psi \rangle } |\langle \psi | U |\psi \rangle |$$.

#### Proof.

Let us first introduce the hypotheses conditioned by the input state $|\psi \rangle$

22 $$\begin{aligned} &H_0^{|\psi \rangle }: \ |\psi \rangle , \\&H_1^{|\psi \rangle }: \ (U \otimes {\mathbbm{1}}) |\psi \rangle . \end{aligned}$$

We do not make any assumptions on the dimension of the auxiliary system for the time being. It will turn out, however, that it suffices if its dimension equals one. The hypotheses in (22) correspond to output states after the application of the extended unitary channel on the state $|\psi \rangle$. For these hypotheses we consider the statistical significance $\delta \in [0,1]$, that is

23 $$\begin{aligned} p_\text {I}^{|\psi \rangle }(\Omega ) = {{\,\text{tr}\,}}\left( ({\mathbbm{1}}-\Omega ) (\Phi _{\mathbbm{1}} \otimes {\mathbbm{1}})(|\psi \rangle \langle \psi |) \right) \le \delta . \end{aligned}$$

Our goal will be to calculate the minimized probability of the type II error

24 $$\begin{aligned} p_\text {II}=\min _{|\psi \rangle }\min _{\Omega : p_{\text {I}}^{|\psi \rangle }(\Omega ) \le \delta } {{\,\text{tr}\,}}(\Omega (\Phi _U \otimes {\mathbbm{1}})(|\psi \rangle \langle \psi |)) =:{{\,\text{tr}\,}}(\Omega _0 (\Phi _U \otimes {\mathbbm{1}})(|\psi _0\rangle \langle \psi _0|)), \end{aligned}$$

where naturally, for the optimal strategy $|\psi _0\rangle$ and $\Omega _0$ it holds that $p_{\text {I}}^{|\psi _0\rangle }(\Omega _0) \le \delta$.

Now we will show that the use of entanglement is unnecessary. From Theorem 1 we know that the probability of the type II error, $p_{\text {II}}$, depends on the minimization of the inner product $\min _{|\psi \rangle } | \langle \psi | U \otimes {\mathbbm{1}} |\psi \rangle |$. Directly from the definition of numerical range we can see that $\langle \psi | U \otimes {\mathbbm{1}} |\psi \rangle \in W(U\otimes {\mathbbm{1}})$. From the property of numerical range given in Eq. (14) and using the notation introduced in Eq. (12) we have

25 $$\begin{aligned} \nu \left( U \otimes {\mathbbm{1}} \right) = \nu \left( U \right) . \end{aligned}$$

Let $|\psi _0\rangle$ be the considered optimal input state, i.e. $|\psi _0\rangle \in \arg \min _{|\psi \rangle } |\langle \psi | U |\psi \rangle |$. Therefore we can reformulate our hypotheses as

26 $$\begin{aligned} &H_0^{|\psi _0\rangle }: \ |\psi _0\rangle , \\&H_1^{|\psi _0\rangle }: \ U |\psi _0\rangle . \end{aligned}$$

These hypotheses, when taking $|\varphi \rangle :=U |\psi _0\rangle$, were the subject of interest in Theorem 1. $\square$

The next corollary follows directly from the above proof.

#### Corollary 2.

*Entanglement is not needed for the certification of unitary channels.*

The following remark states that while considering the input state to the certification scheme, we can restrict our attention to pure states only.

#### Remark 1.

Without loss of generality, we can consider only pure input states. The minimal value of linear objective function

27 $$\begin{aligned} \rho \mapsto {{\,\text{tr}\,}}\left( \Omega \Phi _U(\rho ) \right) \end{aligned}$$

over a convex set $\{\rho \in \mathcal {D}_d: {{\,\text{tr}\,}}\left( \Omega \Phi _{\mathbbm{1}}(\rho ) \right) \ge 1- \delta \}$ is achieved on the extremal points.

### Connection with *q*-numerical range

There exists a close relationship between the above results and the definition of numerical range, which can be seen from the proof of Theorem 2. It the work^15^ the authors show the connection between the discrimination of quantum channels and *q*-numerical range. In this section we show the connection between certification of unitary channels and *q*-numerical range. Recall the definition of *q*-numerical range.

28 $$\begin{aligned} W_q(X) := \{ \langle \psi | X |\varphi \rangle : \langle {\psi }|{\varphi }\rangle =q \}. \end{aligned}$$

Using this notion and the notation introduced in Eq. (12) we can rewrite our results for the probability of the type II error from Theorem 2 as

29 $$\begin{aligned} p_\text {II} = \nu ^2_{\sqrt{1-\delta }}\left( U \otimes {\mathbbm{1}}\right) = \nu ^2_{\sqrt{1-\delta }}\left( U \right) . \end{aligned}$$

An independent derivation of the above formula is presented in Online Appendix B in the Supplementary Materials.

**Figure 1:**
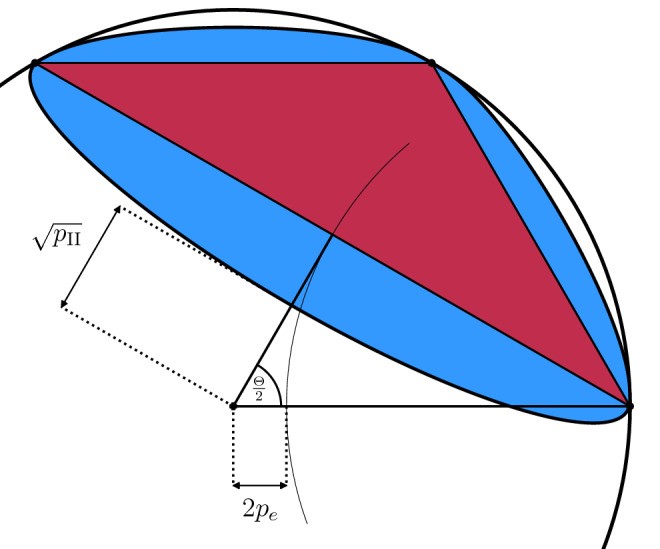
Numerical range *W*(*U*) (red triangle) and $\sqrt{1-\delta }$-numerical range $W_{\sqrt{1-\delta }}(U)$ (blue oval) of $U \in \mathcal {U}_3$ with eigenvalues $1, \text {e}^{ \frac{\pi \text {i}}{3}}$ and $\text {e}^{ \frac{2\pi \text {i}}{3}}$ with statistical significance $\delta = 0.05$. The value $p_e$ is the probability of incorrect symmetric discrimination of channels $\Phi _{\mathbbm{1}}$ and $\Phi _U$.

Let $\Theta$ be the angle between two most distant eigenvalues of a unitary matrix *U*. Then, from the above discussion we can draw a conclusion that for any statistical significance $\delta \in (0,1]$, if $2 \arccos \left( \sqrt{\delta }\right) \le \Theta < \pi$, then although $\Phi _U$ and $\Phi _{\mathbbm{1}}$ cannot be distinguished perfectly, they can be certified with $p_{\text {II}} = 0$. In other words, the numerical range *W*(*U*) does not contain zero but $\sqrt{1-\delta }$-numerical range, $W_{\sqrt{1-\delta }} (U)$, does contain zero. The situation changes when $2 \arccos \left( \sqrt{\delta }\right) > \Theta$. Then, both numerical range *W*(*U*) and $\sqrt{1-\delta }$-numerical range $W_{\sqrt{1-\delta }} (U)$ do not contain zero. This is presented in Fig. 1.

Now we will work towards the construction of the optimal strategy, which will be stated as a corollary. Besides finding the optimal measurement which was shown in previous section we will show a closed-form expression of the optimal input state. For this purpose we will make use of the spectral decomposition of a unitary matrix *U* given by

30 $$\begin{aligned} U = \sum _{i=1}^d \lambda _i |x_i\rangle \langle x_i|. \end{aligned}$$

Let $\lambda _1, \lambda _d$ be a pair of the most distant eigenvalues of *U*. The following corollary is analogous to the corollary from the previous section as it presents the optimal strategy for the certification of unitary channels.

#### Corollary 3.

*By* $|\psi _0\rangle$ *we will denote the optimal state for two-point certification of unitary channels and let* $|\varphi \rangle :=U |\psi _0\rangle$. *Then, the optimal strategy yields*

1. *If* $0 \in W_{\sqrt{1-\delta }}(U)$, *then we have two cases*
  *if* $0 \not \in W(U)$, *then we can take*
    31 $$\begin{aligned} |\psi _0\rangle = \frac{1}{\sqrt{2}} |x_1\rangle + \frac{1}{\sqrt{2}} |x_d\rangle, \end{aligned}$$
    *where* $|x_1\rangle$, $|x_d\rangle$ *are eigenvectors corresponding to the pair of the most distant eigenvalues* $\lambda _1$, $\lambda _d$ *of* *U*. *The optimal measurement is given by* $\Omega _0= |\omega \rangle \langle \omega |$, *where* $|\omega \rangle = \frac{|\widetilde{\omega }\rangle }{|||\widetilde{\omega }\rangle ||}$, $|\widetilde{\omega }\rangle = |\psi _0\rangle - \langle {\varphi }|{\psi _0}\rangle |\varphi \rangle$,
  *if* $0 \in W(U)$, *then we have perfect symmetric distinguishability. Moreover, there exists the probability vector* *p* *such that* $\sum _{i=1}^d \lambda _i p_i = 0$ *and we obtain that*
    32 $$\begin{aligned} |\psi _0\rangle = \sum _{i=1}^d \sqrt{p_i} |x_i\rangle . \end{aligned}$$
    *Analogously, we choose the optimal measurement given by* $\Omega _0= |\omega \rangle \langle \omega |$, *where* $|\omega \rangle = \frac{|{\widetilde{\omega }}\rangle }{|||{\widetilde{\omega }}\rangle ||}$, $|{\widetilde{\omega }}\rangle = |\psi _0\rangle - \langle {\varphi }|{\psi _0}\rangle |\varphi \rangle$. *It easy to see that in this case we have* $\Omega _0 = |\psi _0\rangle \langle \psi _0|$.
2. *If* $0 \not \in W_{\sqrt{1-\delta }}(U)$, *then the discriminator is given by Eq.* (31), *whereas the optimal measurement is can be expressed as* $\Omega _0 = |\omega \rangle \langle \omega |$ *for* $|\omega \rangle = \sqrt{1-\delta } |\psi _0\rangle - \sqrt{\delta } | \psi _0^\perp \rangle$, $|\psi _0^\perp \rangle = \frac{|\widetilde{\psi _0^\perp }\rangle }{|| |\widetilde{\psi _0^\perp } \rangle ||}$, *where* $|\widetilde{\psi _0^\perp }\rangle = |\varphi \rangle - \langle {\psi _0}|{\varphi }\rangle |\psi _0\rangle$.

#### Remark 2.

Observe that the optimal input state $|\psi _0\rangle$ does not depend on $\delta$, while the optimal measurement $\Omega _0$ does depend on the parameter $\delta$ in each case. It is also worth noting that the optimal state in quantum hypothesis testing is of the same form as in the problem of unitary channel discrimination.

## Two-point certification of von Neumann measurements

In this section we will focus on the certification of von Neumann measurements. Recall that every quantum measurement can be associated with a measure-and-prepare quantum channel. Therefore, while studying the certification of quantum measurements we will often take advantage of the certification of quantum channels discussed in the previous section, where we assumed that one of the unitaries was the identity. Similarly, also in the case of certification of von Neumann measurements we will assume that one of the measurements is in the computational basis. Hence, we will be certifying the measurement $\mathcal {P}_{\mathbbm{1}}$ under the alternative hypothesis $\mathcal {P}_U$.

While certifying quantum channels, the most general scenario allows for the use of entanglement by adding an additional system. Hence, in our case of certification of von Neumann measurements, the hypothesis $H_0$ yields that the unknown measurement is $\mathcal {P}_{{\mathbbm{1}}} \otimes {\mathbbm{1}}$ whereas for the alternative hypothesis yields that the measurement is $\mathcal {P}_{U} \otimes {\mathbbm{1}}$.

Now we recall some technical tools which will be used to prove the main result of this work. It was shown in^23^ (Theorem 1) that the diamond norm distance between von Neumann measurements ${\mathcal {P}}_U$ and $\mathcal {P}_{\mathbbm{1}}$ is given by

33 $$\begin{aligned} ||\mathcal {P}_{U} - \mathcal {P}_{\mathbbm{1}}||_\diamond = \min _{E \in {{\mathcal {D}}}{\mathcal {U}}_d} ||\Phi _{UE} - \Phi _{\mathbbm{1}}||_\diamond , \end{aligned}$$

where ${\mathcal {D}}{\mathcal {U}}_d$ is the subgroup of diagonal unitary matrices of dimension *d*. As we can see, the problem of discrimination of von Neumann measurements reduces to the problem of discrimination of unitary channels. From^12^ we know that the diamond norm distance between two unitary channels $\Phi _U$ and $\Phi _{\mathbbm{1}}$ is expressed as

34 $$\begin{aligned} || \Phi _U - \Phi _{\mathbbm{1}} ||_\diamond = 2 \sqrt{1 - \nu ^2\left( U \right) }, \end{aligned}$$

where $$\nu (U) = \min \{ |x|: x \in W(U) \}$$.

### Certification scheme

The scenario of certification of von Neumann measurements is as follows. We prepare some (possibly entangled) input state $|\psi \rangle$ and, as previously, we perform the unknown von Neumann measurement on one part of it. Then, after performing the measurement, the null hypothesis $H_0$ corresponds to the state $\left( \mathcal {P}_{\mathbbm{1}} \otimes {\mathbbm{1}} \right) (|\psi \rangle \langle \psi |)$, while the alternative hypothesis $H_1$ corresponds to the state $\left( \mathcal {P}_U \otimes {\mathbbm{1}} \right) (|\psi \rangle \langle \psi |)$. Our goal is to find an optimal input state and a measurement for which the probability of the type II error is saturated, while the statistical significance $\delta$ is assumed. The results of minimization are stated as the following theorem.

#### Theorem 3.

*Consider the problem of two-point certification of von Neumann measurements with hypotheses*

35 $$\begin{aligned} &H_0: \ \mathcal {P}_{\mathbbm{1}} \otimes {\mathbbm{1}}, \\&H_1: \ \mathcal {P}_U \otimes {\mathbbm{1}}. \end{aligned}$$

*and statistical significance* $\delta \in [0,1]$. *Then, for the most powerful test, the probability of the type II error yields*

36 $$\begin{aligned} p_{\text {II}} = \max _{E \in \mathcal {DU}_d} \nu ^2_{\sqrt{1-\delta }} \left( UE\right) . \end{aligned}$$

It is worth mentioning that we do not make any assumptions on the dimension of the auxiliary system, however its dimension is obviously upper-bounded by the dimension of the input states. Additionally, the dimension of the auxiliary system can be reduced to the Schmidt rank of the input state $|\psi \rangle$^23^ (Proposition 4). It is worth mentioning here that in the certification of von Neumann measurements entanglement can significantly improve the outcome of the protocol while in the case of unitary channel certification it provides no benefit.

In contrast to the certification of unitary channels, the output states $(\mathcal {P}_{\mathbbm{1}} \otimes {\mathbbm{1}})(|\psi \rangle \langle \psi |)$ and $(\mathcal {P}_U \otimes {\mathbbm{1}})(|\psi \rangle \langle \psi |)$ are not necessarily pure. Hence the proof of the Theorem 3 requires more advanced techniques. Luckily, we still can make use of the calculations from  “Certification of unitary channels” section, due to the fact that formally mixed states $(\mathcal {P}_{\mathbbm{1}} \otimes {\mathbbm{1}})(|\psi \rangle \langle \psi |)$ and $(\mathcal {P}_U \otimes {\mathbbm{1}})(|\psi \rangle \langle \psi |)$, conditioned by obtaining the label $i \in \{1,\ldots ,d\}$, are pure.

#### Proof of Theorem 3.

In the scheme of certification of von Neumann measurements the optimized probability of type II error can be expressed as

37 $$\begin{aligned} p_{\text {II}} :=\min _{|\psi \rangle } \min _{\Omega : p_{\text {I}}^{|\psi \rangle }(\Omega ) \le \delta } {{\,\text{tr}\,}}\left( \Omega \left( \mathcal {P}_U \otimes {\mathbbm{1}} \right) (|\psi \rangle \langle \psi |) \right) . \end{aligned}$$

Our goal is to prove that

38 $$\begin{aligned} p_{\text {II}} = \max _{E \in \mathcal {DU}_d} \nu ^2_{\sqrt{1-\delta }} \left( UE\right) . \end{aligned}$$

The proof is divided into two parts. In the first part we will utilize data processing inequality presented in Lemma 1 in Online Appendix C in the Supplementary Materials. Thanks to that, we will show the lower bound for $p_{\text {II}}$. In the second part we will use some technical lemmas presented in Online Appendix C in the Supplementary Materials and we will utilize the results from^23^ to show the upper bound for $p_{\text {II}}$.

#### The lower bound

This part of the proof will mostly be based on data processing inequality. To show that

39 $$\begin{aligned} p_{\text {II}} \ge \max _{E \in \mathcal {DU}_d} \nu ^2_{\sqrt{1-\delta }} \left( UE\right) \end{aligned}$$

let us begin with an observation that every quantum von Neumann measurement $\mathcal {P}_U$ can be rewritten as $\Delta \circ \Phi _{(UE)^\dagger }$, where $\Delta$ denotes the completely dephasing channel and $E \in \mathcal {DU}_d$. Therefore, utilizing data processing inequality in Lemma 1 in Online Appendix C, along with the certification scheme of unitary channels in Theorem 2, the optimized probability of the type II error is lower-bounded by

40 $$\begin{aligned} p_{\text {II}}\ge \min _{|\psi \rangle } \min _{\Omega : p_{\text {I}}^{|\psi \rangle }(\Omega ) \le \delta } {{\,\text{tr}\,}}(\Omega (\Phi _{(UE)^\dagger }\otimes {\mathbbm{1}})(|\psi \rangle \langle \psi |) ) =\nu ^2_{\sqrt{1-\delta }} \left( (UE)^\dagger \right) =\nu ^2_{\sqrt{1-\delta }} \left( UE \right) \end{aligned}$$

which holds for each $E \in \mathcal {DU}_d$. Hence, maximizing the value of $\nu ^2_{\sqrt{1-\delta }} \left( UE \right)$ over $E \in \mathcal {DU}_d$ leads to the lower bound of the form

41 $$\begin{aligned} p_{\text {II}}\ge \max _{E \in \mathcal {DU}_d} \nu ^2_{\sqrt{1-\delta }} \left( UE \right) . \end{aligned}$$

#### The upper bound

Now we proceed to proving the upper bound. The proof of the inequality

42 $$\begin{aligned} p_{\text {II}} \le \max _{E \in \mathcal {DU}_d} \nu ^2_{\sqrt{1-\delta }} \left( UE\right) \end{aligned}$$

will be divided into two cases depending on diamond norm distance between considered measurements $\mathcal {P}_U$ and $\mathcal {P}_{\mathbbm{1}}$. In either case we will construct a strategy, that is choose a state $|\psi _0\rangle$ and a measurement $\Omega _0$. As for every choice of $|\psi \rangle$ and $\Omega$ it holds that

43 $$\begin{aligned} p_{\text {II}} \le {{\,\text{tr}\,}}\left( \Omega (\mathcal {P}_U \otimes {\mathbbm{1}})(|\psi \rangle \langle \psi |)\right) , \end{aligned}$$

we will show that for some fixed $|\psi _0\rangle$ and $\Omega _0$ it holds that

44 $$\begin{aligned} {{\,\text{tr}\,}}\left( \Omega _0 (\mathcal {P}_U \otimes {\mathbbm{1}})(|\psi _0\rangle \langle \psi _0|)\right) = \max _{E \in \mathcal {DU}_d} \nu ^2_{\sqrt{1-\delta }} \left( UE \right) . \end{aligned}$$

First we focus on the case when $\Vert \mathcal {P}_U - \mathcal {P}_{\mathbbm{1}} \Vert _\diamond = 2$. We take a state $|\psi _0\rangle$ for which it holds that

45 $$\begin{aligned} \Vert \mathcal {P}_U - \mathcal {P}_{\mathbbm{1}} \Vert _\diamond = \Vert \left( \left( \mathcal {P}_U - \mathcal {P}_{\mathbbm{1}} \right) \otimes {\mathbbm{1}} \right) (|\psi _0\rangle \langle \psi _0|) \Vert _1. \end{aligned}$$

Then, the output states $(\mathcal {P}_U \otimes {\mathbbm{1}})(|\psi _0\rangle \langle \psi _0|)$ and $(\mathcal {P}_{\mathbbm{1}}\otimes {\mathbbm{1}})(|\psi _0\rangle \langle \psi _0|)$ are orthogonal and by taking the measurement $\Omega _0$ as the projection onto the support of $(\mathcal {P}_{\mathbbm{1}}\otimes {\mathbbm{1}})(|\psi _0\rangle \langle \psi _0|)$ we obtain

46 $$\begin{aligned} {{\,\text{tr}\,}}\left( \Omega _0 (\mathcal {P}_U \otimes {\mathbbm{1}})(|\psi _0\rangle \langle \psi _0|)\right) = 0. \end{aligned}$$

Let us recall that

47 $$\begin{aligned} || \Phi _U - \Phi _{\mathbbm{1}} ||_\diamond = 2 \sqrt{1 - \nu ^2\left( U \right) }, \end{aligned}$$

where $\nu (U) = \min \{ |x|: x \in W(U) \}$ and it holds that^23^

48 $$\begin{aligned} ||\mathcal {P}_{U} - \mathcal {P}_{\mathbbm{1}}||_\diamond = \min _{E \in {\mathcal {D}}{\mathcal {U}}_d} ||\Phi _{UE} - \Phi _1||_\diamond , \end{aligned}$$

where ${\mathcal {D}}{\mathcal {U}}_d$ is the subgroup of diagonal unitary matrices of dimension *d*.

Then, utilizing Eqs. (47) and (48) we obtain that $\max _{E \in \mathcal {DU}_d} \nu ^2\left( UE \right) =0$. Therefore, by the property that $0 \in W_{\sqrt{1-\delta }}(UE)$ whenever $0 \in W(UE)$ (see Online Appendix B), we have that

49 $$\begin{aligned} \max _{E \in \mathcal {DU}_d} \nu ^2_{\sqrt{1-\delta }} \left( UE \right) =0. \end{aligned}$$

Secondly, we consider the situation when $\Vert \mathcal {P}_U - \mathcal {P}_{\mathbbm{1}} \Vert _\diamond < 2$. Let

50 $$\begin{aligned} E_0 \in \underset{E \in \mathcal {DU}_d}{\arg \max } \ \nu \left( UE \right) . \end{aligned}$$

Again, by referring to Eqs. (47) and (48) we obtain that $\nu \left( UE_0 \right) >0$. Let $\lambda _1, \lambda _d$ be a pair of the most distant eigenvalues of $UE_0$. Note that the following relation holds

51 $$\begin{aligned} \nu \left( UE_0 \right) =\frac{|\lambda _1+\lambda _d|}{2}. \end{aligned}$$

As the assumptions of the Lemma 2 in Online Appendix C are saturated for the defined $E_0$, we consider the input state

52 $$\begin{aligned} |\psi _0\rangle =\sum _{i=1}^d \sqrt{\rho _0} |i\rangle \otimes |i\rangle, \end{aligned}$$

where the existence of $\rho _0$ together with its properties are described in Lemma 2 and Corollary 1 in Online Appendix C. Let us define sets

53 $$\begin{aligned} {\mathcal {C}}_i :=\left\{ \Omega : 0 \le \Omega \le {\mathbbm{1}}, \, {{\,\text{tr}\,}}\left( \left( {\mathbbm{1}}- \Omega \right) \frac{\sqrt{\rho _0}|i\rangle \langle i| \sqrt{\rho _0}}{\langle i| \rho _0 |i\rangle }\right) \le \delta \right\} \end{aligned}$$

for each *i* such that $\langle i|\rho |i\rangle \not = 0$. Now we take the measurement $\Omega _0$ as

54 $$\begin{aligned} \Omega _0= \sum _{i=1}^d |i\rangle \langle i| \otimes \Omega _{i}^\top, \end{aligned}$$

where $\Omega _{i}\in {\mathcal {C}}_i$ is defined as

55 $$\begin{aligned} \Omega _i \in \arg \min _{{\widetilde{\Omega }} \in {\mathcal {C}}_i } {{\,\text{tr}\,}}\left( {\widetilde{\Omega }} \frac{\sqrt{\rho _0} U |i\rangle \langle i| U^\dagger \sqrt{\rho _0}}{\langle i| \rho _0 |i\rangle }\right) \end{aligned}$$

for each $i \in \{ 1,\ldots ,d\}$ such that $\langle i|\rho _0|i\rangle \ne 0$ and $\Omega _i = 0$ otherwise.

Now we check that the statistical significance is satisfied, that is for the described strategy we have

56 $$\begin{aligned} p_{\text {I}}^{|\psi _0\rangle }(\Omega _0)=1-{{\,\text{tr}\,}}\left( \Omega _0 (\mathcal {P}_{\mathbbm{1}} \otimes {\mathbbm{1}})(|\psi _0\rangle \langle \psi _0|)\right) =1-\sum _{i=1}^d {{\,\text{tr}\,}}\left( \Omega _{i} \sqrt{\rho _0} |i\rangle \langle i| \sqrt{\rho _0}\right) \le \delta . \end{aligned}$$

Hence, it remains to show that for this setting

57 $$\begin{aligned} {{\,\text{tr}\,}}\left( \Omega _0 (\mathcal {P}_U \otimes {\mathbbm{1}})(|\psi _0\rangle \langle \psi _0|)\right) = \max _{E \in \mathcal {DU}_d} \nu ^2_{\sqrt{1-\delta }}\left( UE \right) . \end{aligned}$$

Direct calculations reveal that

58 $$\begin{aligned} &{{\,\text{tr}\,}}\left( \Omega _0 (\mathcal {P}_U \otimes {\mathbbm{1}})(|\psi _0\rangle \langle \psi _0|)\right) =\sum _{i=1}^d {{\,\text{tr}\,}}\left( \Omega _{i} \sqrt{\rho _0} U|i\rangle \langle i|U^\dagger \sqrt{\rho _0}\right) \\&=\sum _{i=1}^d \langle i| \rho _0 |i\rangle {{\,\text{tr}\,}}\left( \Omega _{i} \frac{\sqrt{\rho _0} U |i\rangle \langle i| U^\dagger \sqrt{\rho _0}}{\langle i| \rho _0 |i\rangle }\right) . \end{aligned}$$

Let us define

59 $$\begin{aligned} p_{\text {II}}^{|i} = {{\,\text{tr}\,}}\left( \Omega _{i} \frac{\sqrt{\rho _0} U |i\rangle \langle i| U^\dagger \sqrt{\rho _0}}{\langle i| \rho _0 |i\rangle }\right) . \end{aligned}$$

Note that due to Corollary 1 in Online Appendix C the absolute value of the inner product between pure states $\frac{\sqrt{\rho _0}|i\rangle }{\Vert \sqrt{\rho _0}|i\rangle \Vert }$ and $\frac{\sqrt{\rho _0} U |i\rangle }{\Vert \sqrt{\rho _0} |i\rangle \Vert }$ is the same for every $i\in \{1,\ldots ,d\}: \langle i| \rho |i\rangle \not = 0$. Therefore we can consider the certification of pure states conditioned on the obtained label *i* with statistical significance $\delta$. From the Theorem 1 we know that $p_\text {II}^{|i}$ depends only on such an inner product between the certified states, hence $p_\text {II}^{|i}=p_\text {II}^{|j}$ for each $i,j: \langle i|\rho |i\rangle , \langle j| \rho |j\rangle \not = 0$. Therefore, we have that the value of $p_\text {II}^{|i}$ will depend on $\left| \frac{\lambda _1 + \lambda _d }{2} \right|$. Thus w.l.o.g. we can assume that $p_{\text {II}}^{|1}\ne 0$ and hence

60 $$\begin{aligned} \sum _{i=1}^d \langle i| \rho _0 |i\rangle p_{\text {II}}^{|i} =p_{\text {II}}^{|1} ={{\,\text{tr}\,}}\left( \Omega _{1} \frac{\sqrt{\rho _0} U |1\rangle \langle 1| U^\dagger \sqrt{\rho _0}}{\langle 1| \rho _0 |1\rangle }\right) \end{aligned}$$

and in the remaining of the proof we will show that

61 $$\begin{aligned} p_{\text {II}}^{|1}= \max _{E \in \mathcal {DU}_d} \nu ^2_{\sqrt{1-\delta }}\left( UE \right) . \end{aligned}$$

It is sufficient to study two cases depending on the relation between $\sqrt{\delta }$ and the inner product

62 $$\begin{aligned} \left| \frac{\langle 1| \rho _0 U |1\rangle }{\langle 1|\rho _0|1\rangle } \right| =\left| \frac{\lambda _1 + \lambda _d }{2} \right| . \end{aligned}$$

In the case when $\left| \frac{\lambda _1 + \lambda _d }{2} \right| \le \sqrt{\delta }$, then due to Theorem 1 we get $p_{\text {II}}^{|1}=0$. On the other hand, we know that $0 \in W_{\sqrt{1-\delta }}(UE_0)$ and hence also

63 $$\begin{aligned} \max _{E \in \mathcal {DU}_d} \nu ^2_{\sqrt{1-\delta }} \left( UE \right) =0. \end{aligned}$$

In the case when $\left| \frac{\lambda _1 + \lambda _d }{2} \right| > \sqrt{\delta }$, then from Theorem 1 we know that

64 $$\begin{aligned} p_{\text {II}}^{|1} = \left( \left| \frac{\lambda _1 + \lambda _d }{2} \right| \sqrt{1-\delta } - \sqrt{1-\left| \frac{\lambda _1 + \lambda _d }{2} \right| ^2} \sqrt{\delta }\right) ^2. \end{aligned}$$

On the other hand, for $E_0 \in \mathcal {DU}_d$ satisfying Eq. (50) we have

65 $$\begin{aligned} \nu ^2_{\sqrt{1-\delta }} \left( UE_0 \right) = \left( \left| \frac{\lambda _1 + \lambda _d }{2} \right| \sqrt{1-\delta } - \sqrt{1-\left| \frac{\lambda _1 + \lambda _d }{2} \right| ^2} \sqrt{\delta }\right) ^2. \end{aligned}$$

By the particular choice of $E_0 \in \mathcal {DU}_d$, this value is equal to $\max _{E \in \mathcal {DU}_d} \nu ^2_{\sqrt{1-\delta }} \left( UE \right)$, hence combining the above equations we finally obtain

66 $$\begin{aligned} p_{\text {II}}^{|1} = \max _{E \in \mathcal {DU}_d} \nu ^2_{\sqrt{1-\delta }} \left( UE \right) . \end{aligned}$$

To sum up, we indicated strategies $\Omega _0$ and $|\psi _0\rangle$ for which the optimized probability of type II error was equal to $\max _{E \in \mathcal {DU}_d} \nu ^2_{\sqrt{1-\delta }} \left( UE \right)$. Combining this with the previously proven inequality

67 $$\begin{aligned} p_{\text {II}}\ge \max _{E \in \mathcal {DU}_d} \nu ^2_{\sqrt{1-\delta }} \left( UE \right) \end{aligned}$$

gives us Eq. (38) and proves that the proposed strategy $|\psi _0\rangle , \Omega _0$ is optimal. $\square$

##### Remark 3.

Similarly to the case of unitary channel certification, the optimal input state $|\psi _0\rangle$ does not depend on $\delta$, while the optimal measurement $\Omega _0$ does depend on $\delta$. Moreover, the optimal state has the same form as in the problem of discrimination of von Neumann measurements.

Finally, in Algorithm 1 we present a protocol which describes the optimal certification strategy based on the proof of Theorem 3. 
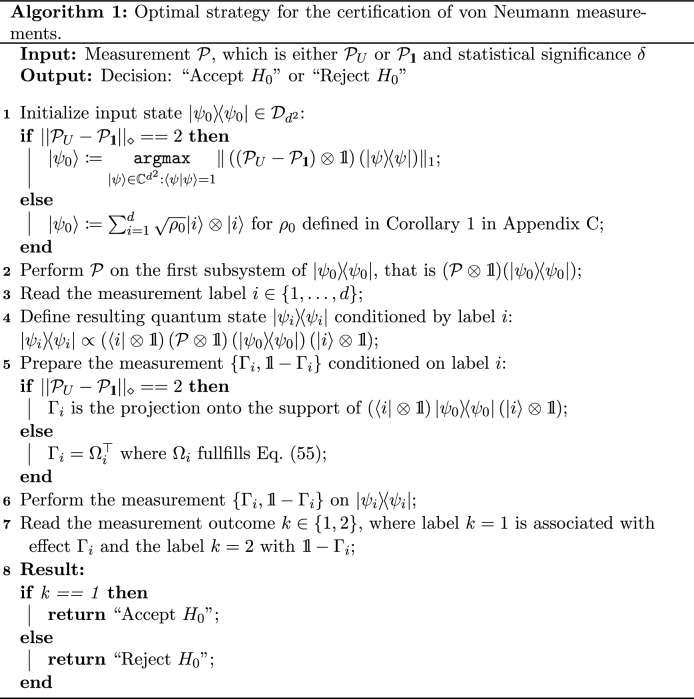


Below, we provide a simple example of application of Algorithm 1 in certification of a measurement performed in the Hadamard basis.

##### Example 1.

We will certify von Neumann measurements ${\mathcal {P}}_{\mathbb {H}}$ and $\mathcal {P}_{{\mathbbm{1}}}$, where ${\mathbb {H}}$ is the Hadamard matrix.

1. We calculate the distance between $\mathcal {P}_{{\mathbbm{1}}}$ and ${\mathcal {P}}_{\mathbb {H}}$. Using semidefinite programming^23,38^ we obtain $|| {\mathcal {P}}_{{\mathbb {H}}} - \mathcal {P}_{\mathbbm{1}}||_\diamond = \sqrt{2}$. Observe that matrix $E_0$ minimizing (48) is of the form $$E_0 = \frac{1}{\sqrt{2}} \left( \begin{array}{cc} 1+i&{}0 \\ 0&{}-1-i \end{array} \right)$$, which means
  68 $$\begin{aligned} ||\mathcal {P}_{{\mathbb {H}}} - \mathcal {P}_{\mathbbm{1}}||_\diamond = ||\Phi _{{\mathbb {H}}E_0} - \Phi _{\mathbbm{1}}||_\diamond . \end{aligned}$$
  In order to construct $\rho _0$ we use Lemma 2 in the Supplementary Materials. There exist states $\rho _1, \rho _2$ of the form $$\rho _1 = \frac{1}{2}\left( \begin{array}{cc} 1&{}i \\ -i&{}1 \end{array} \right)$$ and $$\rho _2 = \frac{1}{2} \left( \begin{array}{cc} 1&{}-i \\ i&{}1 \end{array} \right)$$. Thus, from Corollary 1 in the Supplementary Materials we have
  69 $$\begin{aligned} \rho _0 = \frac{1}{2}(\rho _1 + \rho _2) = \frac{1}{2} \left( \begin{array}{cc} 1&{}0 \\ 0&{}1 \end{array} \right) . \end{aligned}$$
  Hence, the input state $|\psi _0\rangle$ has a form
  70 $$\begin{aligned} |\psi _0\rangle :=\sum _{i=1}^2 \frac{1}{\sqrt{2}} |i\rangle \otimes |i\rangle . \end{aligned}$$
2. We perform $\mathcal {P}$ on the first subsystem of $|\psi _0\rangle \langle \psi _0|$, that is $(\mathcal {P}\otimes {\mathbbm{1}}) (|\psi _0\rangle \langle \psi _0|)$.
3. We read the measurement label either $i= 1$ or $i = 2$.
4. We reduce our problem to certification between states $|\psi _i\rangle = {\mathbb {H}} |i\rangle$ or $|\psi _i\rangle = |i\rangle$.
5. According to the optimal strategy for two-point certification of pure quantum states (Corollary 1), we prepare conditional measurements $\Gamma _i$. For a fixed statistical significance $\delta$ and a given label *i*, the optimal measurement $\Gamma _i$ is defined as
  1. if $$\delta \ge \frac{1}{2}$$, then $$\Gamma _i = {\mathbb {H}} |i^\perp \rangle \langle i^\perp | {\mathbb {H}}$$.
  2. if $\delta < \frac{1}{2}$, then $\Gamma _i = |\gamma \rangle \langle \gamma |$ for $|\gamma \rangle = \sqrt{1-\delta } |i\rangle - \sqrt{\delta } | i^\perp \rangle$.
6. We perform the measurement $\{ \Gamma _i, {\mathbbm{1}} - \Gamma _i \}$ on $|\psi _i\rangle \langle \psi _i|$.
7. We read the measurement outcome $k \in \{1,2\}$.
8. Finally, basing on value of *k* we make a decision whether we accept (for $k = 1$) or reject (for $k = 2$) the null hypothesis $H_0$.

## Parallel multiple-shot certification

In this section we focus on the scenarios in which we have access to *N* copies of the certified quantum objects. The copies of a given object can be used in many configurations. The most general strategy, in the literature referred to as the adaptive scenario, assumes that we are allowed to perform any processing between uses of each provided copy. The adaptive scenario can be described with the formalism of quantum networks^39^. In this paper we restrict our attention only to the special case of adaptive strategy, when all copies are used in parallel. This approach, known as the parallel scenario, has a simplified description based on the tensor product of the copies of a given quantum object. Henceforth, in our cases, the tensor product of pure states will be again a pure state, tensor product of unitary channels—a unitary channel and tensor product of von Neumann measurements—a von Neumann measurement. Therefore, we will be able to apply our results from previous sections.

At this point, a natural question arises: can the parallel scenario be optimal? In the case of discrimination of unitary channels and von Neumann measurements, the positive answer was obtained^24,40^. Nevertheless, in some special cases of quantum channels or quantum measurements by using an adaptive scenario we are able to improve the probability of correct discrimination of such objects^20,21^. In the case of certification of quantum objects, the optimality of parallel scenario was showed for unitary channels^27^. In this section, we prove the parallel approach for certification of von Neumann measurements is also optimal, see Theorem 5.

Let us begin with the certification of pure states. Such certification can be understood as certifying states $|\psi \rangle ^{\otimes N}$ and $|\varphi \rangle ^{\otimes N}$. The following corollary generalizes the results from Theorem 1.

### Corollary 4.

*In the case of certification of pure states* $|\psi \rangle ^{\otimes N}$ *and* $|\varphi \rangle ^{\otimes N}$ *with statistical significance* $\delta \in [0,1]$, *the minimized probability of the type II error yields*

71 $$\begin{aligned} p_{\text {II}}^{(N)} = \left\{ \begin{array}{ll} 0 &{} |\langle {\psi }|{\varphi }\rangle |^N \le \sqrt{\delta } \\ \left( |\langle {\psi }|{\varphi }\rangle |^N \sqrt{1-\delta } - \sqrt{1-|\langle {\psi }|{\varphi }\rangle |^{2N} } \sqrt{\delta }\right) ^2 &{} |\langle {\psi }|{\varphi }\rangle |^N > \sqrt{\delta }, \end{array} \right. \end{aligned}$$

*where* *N* *is the number of uses of the pure state.*

One can note that for a given statistical significance $\delta$, by taking $N \ge \frac{\log \sqrt{\delta }}{\log |\langle {\psi }|{\varphi }\rangle |}$ we obtain $p_\text {II}=0$. This is not in contradiction with the statement that if one cannot distinguish states perfectly in one step, then they cannot by distinguished perfectly in any finite number of tries, because the error is hidden in $p_\text {I}$. This error decays exponentially, and the optimal exponential error rate, depending on a formulation, can be stated as the Stein bound, the Chernoff bound, the Hoeffding bound, and the Han–Kobayashi bound, see^41^ and references therein.

Secondly, we focus on the certification of unitary channels. The scenario of parallel certification can be seen as certifying channels $\Phi _{{\mathbbm{1}}^{\otimes N}}$ and $\Phi _{U^{\otimes N}}$. Hence, for the parallel certification of such unitary channels we have the following corollary generalizing the results from Theorem 2.

### Corollary 5.

*In the case of parallel certification of unitary channels* $\Phi _{{\mathbbm{1}}^{\otimes N}}$ *and* $\Phi _{U^{\otimes N}}$ *with statistical significance* $\delta \in [0,1]$, *the minimized probability of the type II error yields*

72 $$\begin{aligned} p_{\text {II}}^{(N)} = \nu ^2_{\sqrt{1-\delta }} \left( U^{\otimes N}\right), \end{aligned}$$

*where* *N* *is the number of uses of the unitary channel.*

**Figure 2:**
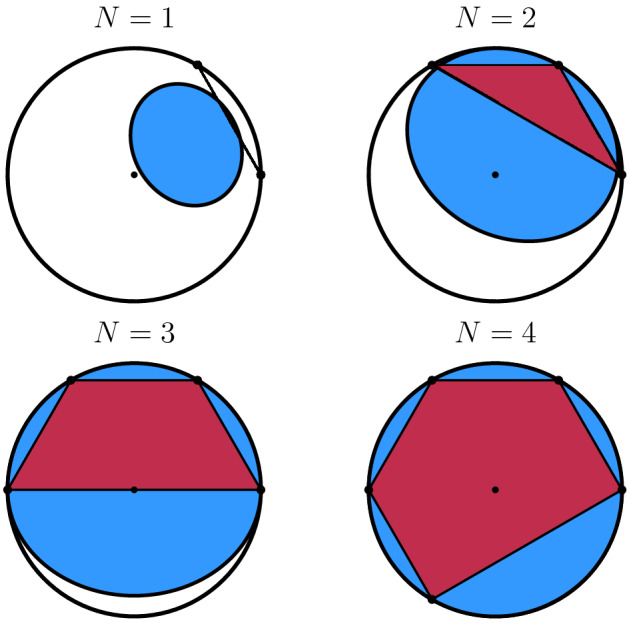
Numerical ranges $W(U^{\otimes N})$ (polytopes) and $\sqrt{1-\delta }$-numerical ranges $W_{\sqrt{1-\delta }}(U^{\otimes N})$ (ovals) of $U \in \mathcal {U}_2$ with eigenvalues 1 and $\text {e}^{ \frac{\pi \text {i}}{3}}$, for $N=1,2,3,4$ with statistical significance $\delta = 0.7$.

From the above it follows that if $0 \in W_{\sqrt{1- \delta }}(U^{\otimes N})$, then the channels $\Phi _{\mathbbm{1}}^{\otimes N}$ and $\Phi _U^{\otimes N}$ can be certified with $p_{\text {II}} = 0$. Let $\Theta$ be the angle between a pair of two most distant eigenvalues of a unitary matrix *U*. The perfect certification can be achieved by taking $N = \lceil \frac{2\arccos \sqrt{\delta }}{\Theta } \rceil$. Observe that in the special case $\delta = 0$, we recover the well-known formula $N = \lceil \frac{ \pi }{\Theta } \rceil$ being the number of unitary channels required for perfect discrimination in the scheme of symmetric distinguishability of unitary channels^17^. The dependence between the number *N* of used unitary channels and the shape of $W_{\sqrt{1-\delta }}(U^{\otimes N})$ is presented in Fig. 2.

We have established similar results for the case of certifying *N* copies of von Neumann measurements $\mathcal {P}_{\mathbbm{1}}$ and $\mathcal {P}_U$. We consider only the parallel scenario and therefore this can be understood as certifying von Neumann measurements $\mathcal {P}_{U^{\otimes N}}$ and $\mathcal {P}_{{\mathbbm{1}}^{\otimes N}}$. This issue is studied in Theorem 4. Moreover, it will turn out in Theorem 5 that the parallel scenario is optimal for the certification of von Neumann measurements.

### Theorem 4.

*In the case of certification of von Neumann measurements* $\mathcal {P}_{U^{\otimes N}}$ and $\mathcal {P}_{{\mathbbm{1}}^{\otimes N}}$ *with statistical significance* $\delta \in [0,1]$, *the minimized probability of the type II error yields*

73 $$\begin{aligned} p_{\text {II}}^{(N)} = \max _{E \in \mathcal {DU}_{d}} \nu ^2_{\sqrt{1-\delta }} \left( U^{\otimes N}E^{\otimes N}\right) , \end{aligned}$$

*where* *N* *is the number of uses of the von Neumann measurements.*

### Proof.

The von Neumann measurements $\mathcal {P}_{{\mathbbm{1}}^{\otimes N}}$ and $\mathcal {P}_{U^{\otimes N}}$ satisfy assumptions of Theorem 3, therefore we have

74 $$\begin{aligned} p_{\text {II}}^{(N)} = \max _{E \in \mathcal {DU}_{d^N}} \nu ^2_{\sqrt{1-\delta }} \left( U^{\otimes N}E\right) . \end{aligned}$$

Whereas, the equality

75 $$\begin{aligned} \max _{E \in \mathcal {DU}_{d^N}} \nu ^2_{\sqrt{1-\delta }} \left( U^{\otimes N}E\right) = \max _{E \in \mathcal {DU}_{d}} \nu ^2_{\sqrt{1-\delta }} \left( U^{\otimes N}E^{\otimes N}\right) \end{aligned}$$

follows from^24^ (Theorem 1). $\square$

Finally, we state the theorem providing the optimality of parallel scenario for the certification of von Neumann measurements.

### Theorem 5.

*The parallel scenario for certification of von Neumann measurements is optimal. More formally, for any adaptive certification scenario, the probability of the type II error cannot be smaller than in the parallel scenario.*

### Proof.

Let us denote by $\Xi (\cdot )$ an adaptive scenario, whose input are *N* copies of a given quantum operation and outputs a quantum state. Let $\tilde{p}_{\text {II}}^{(N)}$ be the minimized probability of the type II error for states $\Xi \left( \mathcal {P}_{\mathbbm{1}}^{(N)}\right) , \Xi \left( \mathcal {P}_U^{(N)}\right)$. Define $d_1 \le d_2 \le \ldots \le d_N$ to be a non-decreasing sequence of natural numbers and assume that $d_N = d_N' d_N''$ for $d_N', d_N'' \in \mathbb {N}$. The numbers $d_1,\ldots ,d_N$ will denote sizes of auxiliary systems occurring in a construction of $\Xi \left( \mathcal {P}_{\mathbbm{1}}^{(N)}\right) , \Xi \left( \mathcal {P}_U^{(N)}\right)$. We assume that the last auxiliary system having dimension $d_N$ is a tensor product of two subsystems: one with dimension $d_N'$ and second with dimension $d_N''$, which will be traced out. The general adaptive scenario for certification of *N* copies of von Neumann measurements $\mathcal {P}_U$, $\mathcal {P}_{\mathbbm{1}}$ can be represented as^24^:

76 $$\begin{aligned} \Xi \left( \mathcal {P}_U^{(N)}\right) =&({\mathbbm{1}}_{d^{N-1}} \otimes \mathcal {P}_U \otimes {\mathbbm{1}}_{d_N'} \otimes {{\,\text{tr}\,}}_{d_N''}) \circ \Xi _{N-1} \circ \ldots \circ ({\mathbbm{1}}_d \otimes \mathcal {P}_U \otimes {\mathbbm{1}}_{d^{N-2}d_2}) \circ \\&\Xi _1 \circ (\mathcal {P}_U \otimes {\mathbbm{1}}_{d^{N-1}d_1})\left( |\psi _0\rangle \langle \psi _0|\right) , \\ \Xi \left( \mathcal {P}_{\mathbbm{1}}^{(N)}\right) =&({\mathbbm{1}}_{d^{N-1}} \otimes \mathcal {P}_{\mathbbm{1}} \otimes {\mathbbm{1}}_{d_N'} \otimes {{\,\text{tr}\,}}_{d_N''}) \circ \Xi _{N-1} \circ \ldots \circ ({\mathbbm{1}}_d \otimes \mathcal {P}_{\mathbbm{1}} \otimes {\mathbbm{1}}_{d^{N-2}d_2}) \circ \\&\Xi _1 \circ (\mathcal {P}_{\mathbbm{1}} \otimes {\mathbbm{1}}_{d^{N-1}d_1})\left( |\psi _0\rangle \langle \psi _0|\right) . \end{aligned}$$

**Figure 3:**
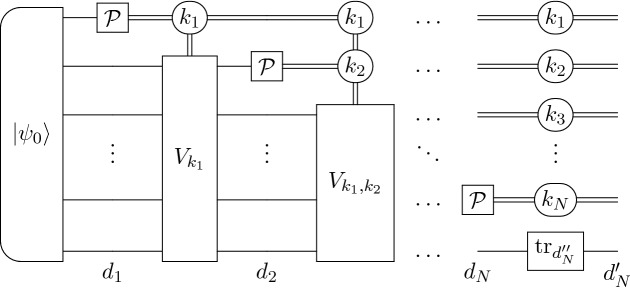
Application of an adaptive scenario $\Xi \left( \cdot \right)$ on *N* copies of a von Neumann measurement $\mathcal {P}$ where $\mathcal {P}\in \{\mathcal {P}_{\mathbbm{1}}, \;\mathcal {P}_U\}$.

The channels $\Xi _i$ are given by $\Xi _i(X) = W_i X W_i^\dagger$, such that

77 $$\begin{aligned} W_i = \sum _{k_1,k_2, \ldots , k_i} |k_1, k_2,\ldots , k_i\rangle \langle k_1, k_2,\ldots , k_i| \otimes V_{k_1, k_2, \ldots ,k_i}, \end{aligned}$$

where $V_{k_1, k_2, \ldots ,k_i} \in M_{d^{N-i} d_i, d^{N-i} d_{i+1}}$ are isometry matrices for each $i \in \{1,\ldots ,N-1\}$. The above scenario is presented in Fig. 3.

**Figure 4:**
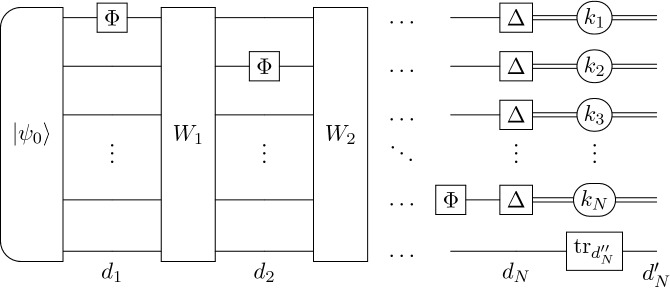
An equivalent description of Eq. (76), formalized as Eq. (78). The scenario $\Xi (\cdot )$ is applied to *N* copies of unitary channel $\Phi$, where $\Phi \in \{\Phi _{\mathbbm{1}}, \Phi _{(UE_0)^\dagger }\}$. As a last step we perform a completely dephasing channel $\Delta$.

Let us decompose a von Neumann measurement $\mathcal {P}_{U}$ as a composition of a unitary channel $\Phi _{(UE_0)^\dagger }$ and the completely dephasing channel $\Delta$, that is $\mathcal {P}_U = \Delta \circ \Phi _{(UE_0)^\dagger }$, where $E_0$ is a matrix maximizing Eq. (73). Therefore, one can rewrite the adaptive scenarios $\Xi \left( \mathcal {P}_{U}^{(N)}\right)$ and $\Xi \left( \mathcal {P}_{\mathbbm{1}}^{(N)}\right)$ as (see also Fig. 4)

78 $$\begin{aligned} \Xi \left( \mathcal {P}_{U}^{(N)}\right)&= \left( \Delta ^{\otimes N} \otimes {\mathbbm{1}}_{d_N'} \otimes {{\,\text{tr}\,}}_{d_N''}\right) \circ \Xi \left( \Phi _{(UE_0)^\dagger }^{(N)}\right) ,\\ \Xi \left( \mathcal {P}_{{\mathbbm{1}}}^{(N)}\right)&= \left( \Delta ^{\otimes N} \otimes {\mathbbm{1}}_{d_N'} \otimes {{\,\text{tr}\,}}_{d_N''}\right) \circ \Xi \left( \Phi _{{\mathbbm{1}}}^{(N)}\right) , \end{aligned}$$

where

79 $$\begin{aligned} \Xi \left( \Phi _{(UE_0)^\dagger }^{(N)}\right) =&({\mathbbm{1}}_{d^{N-1}} \otimes \Phi _{(UE_0)^\dagger } \otimes {\mathbbm{1}}_{d_N}) \circ \Xi _{N-1} \circ \ldots \circ ({\mathbbm{1}}_d \otimes \Phi _{(UE_0)^\dagger } \otimes {\mathbbm{1}}_{d^{N-2}d_2}) \circ \\&\Xi _1 \circ (\Phi _{(UE_0)^\dagger } \otimes {\mathbbm{1}}_{d^{N-1}d_1})\left( |\psi _0\rangle \langle \psi _0|\right) , \\ \Xi \left( \Phi _{{\mathbbm{1}}}^{(N)}\right) =&({\mathbbm{1}}_{d^{N-1}} \otimes \Phi _{{\mathbbm{1}}} \otimes {\mathbbm{1}}_{d_N}) \circ \Xi _{N-1} \circ \ldots \circ ({\mathbbm{1}}_d \otimes \Phi _{{\mathbbm{1}}} \otimes {\mathbbm{1}}_{d^{N-2}d_2}) \circ \\&\Xi _1 \circ (\Phi _{{\mathbbm{1}}} \otimes {\mathbbm{1}}_{d^{N-1}d_1})\left( |\psi _0\rangle \langle \psi _0|\right) . \end{aligned}$$

Let us observe that the scenarios $\Xi \left( \Phi _{(UE_0)^\dagger }^{(N)}\right) , \Xi \left( \Phi _{{\mathbbm{1}}}^{(N)}\right)$ describe certification of *N* copies of unitary channels $\Phi _{(UE_0)^\dagger }$ and $\Phi _{{\mathbbm{1}}}$. Using the data processing inequality in Lemma 1 in Supplementary Materials, the probability of the type II error for certification between $\Xi \left( \mathcal {P}_{U}^{(N)}\right)$ and $\Xi \left( \mathcal {P}_{{\mathbbm{1}}}^{(N)}\right)$ is no smaller that for $\Xi \left( \Phi _{(UE_0)^\dagger }^{(N)}\right)$ and $\Xi \left( \Phi _{{\mathbbm{1}}}^{(N)}\right)$.

Following^27^, the minimized probability of the type II error for *N*-copies of unitary channels $\Phi _{(UE_0)^\dagger }$ and $\Phi _{{\mathbbm{1}}}$ is achieved in the parallel scenario, that is whenever $\Xi \left( \Phi _{(UE_0)^\dagger }^{(N)}\right) = \Phi _{(UE_0)^\dagger }^{\otimes N} (|\psi _0\rangle \langle \psi _0|)$ and $\Xi \left( \Phi _{{\mathbbm{1}}}^{(N)}\right) =\Phi _{\mathbbm{1}}^{\otimes N} (|\psi _0\rangle \langle \psi _0|)$, where $|\psi _0\rangle$ is an optimal state. For this case, from Corollary 5 the probability of the type II error for certification of *N* copies of unitary channels $\Phi _{(UE_0)^\dagger }$ and $\Phi _{{\mathbbm{1}}}$ is equal $\nu ^2_{\sqrt{1-\delta }} \left( U^{\otimes N}E_0^{\otimes N}\right)$. Hence, we obtain

80 $$\begin{aligned} {{\tilde{p}}}_{\text {II}}^{(N)} \ge \nu ^2_{\sqrt{1-\delta }} \left( U^{\otimes N}E_0^{\otimes N}\right) . \end{aligned}$$

From Theorem 4, we have

81 $$\begin{aligned} p_{\text {II}}^{(N)} = \nu ^2_{\sqrt{1-\delta }} \left( U^{\otimes N}E_0^{\otimes N}\right) , \end{aligned}$$

where $p_{\text {II}}^{(N)}$ is the minimized probability of type II error for the parallel certification of von Neumann measurements $\mathcal {P}_{\mathbbm{1}}$ and $\mathcal {P}_U$. Then, we have

82 $$\begin{aligned} {\tilde{p}}_{\text {II}}^{(N)} \ge p_{\text {II}}^{(N)} \end{aligned}$$

which finishes the proof. $\square$

## Conclusions

In this work we studied the two-point certification of quantum states, unitary channels and von Neumann measurements. The problem of certification of quantum objects is inextricably related with quantum hypothesis testing. We were interested in minimizing the probability of type II error given the upper bound on the probability of type I error.

Although the problems of certification of quantum states and unitary channels are well-studied, we pointed out the connection of certification of unitary channels with the notion of *q*-numerical range. Afterwards, we extended this approach to the certification of von Neumann measurements and found a formula for minimized probability of the type II error and the optimal certification strategy. It turned out that this formula can be also connected with the notion of *q*-numerical range. Remarkably, it appeared that in the case of certification of von Neumann measurements the use of entangled input state can significantly improve the certification.

Finally, we focused on the certification of the von Neumann measurements in the parallel scenario. More precisely, we generalized the above results for the situation when the von Neumann measurements can be used *N* times in parallel. We showed that optimal certification of von Neumann measurements can be performed without any additional processing, i.e. in the parallel way.

## Supplementary Information

Supplementary Information 1.

Supplementary Information 2.
