# Author Correction: On the optimal certification of von Neumann measurements

**DOI:** 10.1038/s41598-022-10219-7

**Published:** 2022-04-07

**Authors:** Paulina Lewandowska, Aleksandra Krawiec, Ryszard Kukulski, Łukasz Pawela, Zbigniew Puchała

**Affiliations:** 1 Institute of Theoretical and Applied Informatics, Polish Academy of Sciences, ul. Bałtycka 5, 44-100 Gliwice, Poland; 2 Faculty of Physics, Astronomy and Applied Computer Science, Jagiellonian University, ul. Łojasiewicza 11, 30-348 Kraków, Poland

Correction to: *Scientific Reports* 10.1038/s41598-021-81325-1, published online 11 February 2021

The original version of this Article contained an error in the Acknowledgements section.

“This work was supported by the Foundation for Polish Science (FNP) under Grant Number POIR.04.04.00-00-17C1/18-00.”

now reads:

“The project “Near-term quantum computers Challenges, optimal implementations and applications” under Grant Number POIR.04.04.00-00-17C1/18-00, is carried out within the Team-Net programme of the Foundation for Polish Science co-financed by the European Union under the European Regional Development Fund.”

The original Article has been corrected.

