# Supplementary material for: On the optimal certification of von Neumann measurements
Source: Sci Rep. 2021 Feb 11;11:3623. doi: 10.1038/s41598-021-81325-1 (PMC7878518; doi:10.1038/s41598-021-81325-1)
Supplement: Supplementary file 1 — Supplementary Information 1. [file 41598_2021_81325_MOESM1_ESM.pdf]

ON THE OPTIMAL CERTIFICATION OF VON NEUMANN MEASUREMENTS – SUPPLEMENTARY MATERIALS

PAULINA LEWANDOWSKA[1], ALEKSANDRA KRAWIEC[1], RYSZARD KUKULSKI[1], ŁUKASZ PAWELA* [1], AND ZBIGNIEW PUCHAŁA[1,2]

[1] *Institute of Theoretical and Applied Informatics, Polish Academy of Sciences, ul. Bałtycka 5, 44-100 Gliwice, Poland*

[2] *Faculty of Physics, Astronomy and Applied Computer Science, Jagiellonian University, ul. Łojasiewicza 11, 30-348 Kraków, Poland*

* E-mail address: `lpawela@iitis.pl`

Appendix A. Certification of states

In this appendix we present the proof of Theorem 1.

Proof of Theorem 1. Without loss of generality we can assume that $|\varphi\rangle = \alpha|\psi\rangle + \beta|\psi^\perp\rangle$, for some $\alpha, \beta \geq 0$ satisfying $\alpha^2+\beta^2 = 1$. For any effect $\widetilde{\Omega}$ satisfying $\langle\psi|\widetilde{\Omega}|\psi\rangle \geq 1-\delta$, the effect Ω defined as $\Omega = \Pi\widetilde{\Omega}\Pi$, where $\Pi = |\psi\rangle\langle\psi| + |\psi^\perp\rangle\langle\psi^\perp|$, also satisfies the condition $\langle\psi|\Omega|\psi\rangle \geq 1-\delta$ and simultaneously returns the same value of probability of type II error. Hence, we can assume that rank-2 operator Ω satisfies $\Omega = \Pi\Omega\Pi$. From the above, let $\Omega = a\Pi + b|\omega\rangle\langle\omega|$, where $|\omega\rangle = c|\psi\rangle - d|\psi^\perp\rangle$, $c \geq 0$, $d \in \mathbb{C}$, such that $c^2 + |d|^2 = 1$ and $a, b \in [0,1]$, such that $a+b \leq 1$. By the assumption on the value p_{I}, we have

$$1 - p_{\mathrm{I}}(\Omega) = \langle\psi|\Omega|\psi\rangle = a + bc^2 \geq 1-\delta. \tag{1}$$

Let us calculate the probability p_{II}:

$$p_{\mathrm{II}} = \min_{\Omega: p_{\mathrm{I}}(\Omega)\leq\delta} \langle\varphi|\Omega|\varphi\rangle = \min_{a,b,c,d\in\mathcal{A}} \left(\alpha^2(a+bc^2) + \beta^2(a+b|d|^2) - 2\alpha\beta bc\Re(d)\right) \tag{2}$$

where $\mathcal{A} := \{a,b,c,d : a+b \leq 1,\ a+bc^2 \geq 1-\delta,\ c^2+|d|^2 = 1,\ a,b,c \in [0,1],\ d \in \mathbb{C}\}$. Note that the above formula is minimized when $d \in \mathbb{R}$ is nonnegative. Hence

$$\langle\varphi|\Omega|\varphi\rangle = a + b\left(\alpha c - \beta d\right)^2. \tag{3}$$

Thus, our task reduces to minimizing the formula

$$p_{\mathrm{II}} = \min_{a,b,c\in\mathcal{B}} a + b\left(\alpha c - \beta\sqrt{1-c^2}\right)^2 \tag{4}$$

where $\mathcal{B} := \{a,b,c \in [0,1],\ a+b \leq 1,\ a+bc^2 \geq 1-\delta\}$. We consider two cases.

(1) If $\alpha \leq \sqrt{\delta}$, then we take $a = 0, b = 1, c = \beta, d = \sqrt{1-\beta^2}$. In this case $a,b,c \in \mathcal{B}$ and we obtain $p_{\mathrm{II}} = 0$. The optimal strategy is represented by effect $\Omega_0 = |\omega\rangle\langle\omega|$, where $|\omega\rangle = \beta|\psi\rangle - \alpha|\psi^\perp\rangle$.

(2) Let $\alpha > \sqrt{\delta}$ and take $a = 0, b = 1, c = \sqrt{1-\delta}, d = \sqrt{\delta}$. Again $a, b, c \in \mathcal{B}$ and $p_{\mathrm{II}} = \left(\alpha\sqrt{1-\delta} - \beta\sqrt{\delta}\right)^2$. The optimal strategy is represented by effect $\Omega_0 = |\omega\rangle\langle\omega|$ where $|\omega\rangle = \sqrt{1-\delta}|\psi\rangle - \sqrt{\delta}|\psi^\perp\rangle$. The optimality of this value can be checked by using standard constrained optimization techniques.

□

Appendix B. q-numerical range and certification of unitary channels

B.1. q-numerical range in the problem of two-point certification of unitary channels. In this appendix we will present an alternative derivation the result for the probability of the type II error in the certification of unitary channels given in Eq. (30).

We would like to bound the probability of the type I error by δ, that is $p_{\mathrm{I}}^{|\psi\rangle}(\Omega) = \operatorname{tr}((\mathbb{1} - \Omega)|\psi\rangle\langle\psi|) \leq \delta$. Let us consider $\Omega = |\omega\rangle\langle\omega|$. Hence, we have

$$\operatorname{tr}\left(\Omega|\psi\rangle\langle\psi|\right) = |\langle\omega|\psi\rangle|^2 \geq 1 - \delta. \tag{5}$$

The probability of the type II error takes the form

$$\begin{aligned} p_{\mathrm{II}} &= \min_{|\psi\rangle} \min_{\Omega: p_{\mathrm{I}}^{|\psi\rangle}(\Omega) \leq \delta} \operatorname{tr}\left(\Omega(U \otimes \mathbb{1})|\psi\rangle\langle\psi|(U^\dagger \otimes \mathbb{1})\right) \\ &= \min_{|\psi\rangle} \min_{|\omega\rangle: p_{\mathrm{I}}^{|\psi\rangle}(|\omega\rangle\langle\omega|) \leq \delta} \langle\psi|(U^\dagger \otimes \mathbb{1})|\omega\rangle\langle\omega|(U \otimes \mathbb{1})|\psi\rangle \\ &= \min_{|\psi\rangle} \min_{|\omega\rangle: p_{\mathrm{I}}^{|\psi\rangle}(|\omega\rangle\langle\omega|) \leq \delta} |\langle\psi|(U \otimes \mathbb{1})|\omega\rangle|^2. \end{aligned} \tag{6}$$

Let us recall that the q-numerical range is defined as

$$W_q(A) = \{\langle\xi_0|A|\xi_1\rangle : \langle\xi_0|\xi_1\rangle = q\} \tag{7}$$

and we use the notation

$$\nu_q(X) = \min\{|x| : x \in W_q(X)\}. \tag{8}$$

Now from the definition of the q-numerical range for $q = \sqrt{1-\delta}$ and its properties [1]

$$W_{q'} \subseteq \frac{q'}{q} W_q \quad \text{for} \quad q \leq q', \quad q, q' \in \mathbb{R} \tag{9}$$

and

$$W_q(X \otimes \mathbb{1}) = W_q(X), \quad q \in \mathbb{R} \tag{10}$$

it easy to see that

$$\nu_q(X \otimes \mathbb{1}) = \nu_q(X), \quad q \in \mathbb{R}, \tag{11}$$

which will imply that

$$p_{\mathrm{II}} = \nu^2_{\sqrt{1-\delta}}\left(U \otimes \mathbb{1}\right) = \nu^2_{\sqrt{1-\delta}}\left(U\right). \tag{12}$$

Therefore, we conclude that the use of entanglement for the case of certification of unitary channels does not improve the certification.

B.2. **Distance of q-numerical range to zero.** In this subsection we will focus on calculating the distance from the q-numerical range the to the origin of the coordinate system. Let us begin with the two-dimensional case when the unitary matrix U has two eigenvalues λ_1 and λ_2. Without loss of generality we can assume $\lambda_1 = 1$. From [2] we know that the q-numerical range is an elliptical disc with eccentricity equal to q and foci $q\lambda_1$ and $q\lambda_2$, see Fig 1. Let c denote the distance from the center of the ellipse to the focus and a be the distance from the center of the ellipse to its vertex. Using this notation the eccentricity yields $q = c/a$. Let b denote the distance from the center of the ellipse to its co-vertex, which it the point which saturates the minimum.

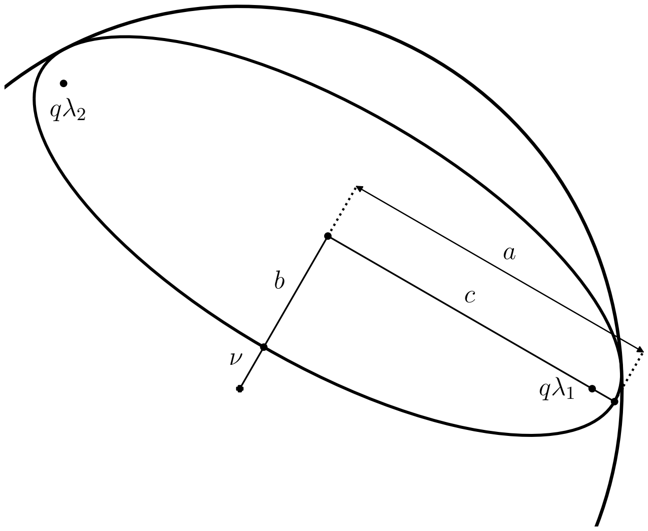

FIGURE 1. Schematic illustration of an ellipse and notation used in Appendix, where we use shortcut notation $\nu := \nu_q(U)$.

First, we will calculate b. We note that

$$c = \frac{1}{2} \|q\lambda_1 - q\lambda_2\| = \frac{q}{2} \|\lambda_1 - \lambda_2\| = \frac{\sqrt{1-\delta}}{2} \|\lambda_1 - \lambda_2\| . \tag{13}$$

From the properties of the ellipse and the form of the eccentricity q we have

$$b = \sqrt{a^2 - c^2} = \sqrt{\frac{c^2}{q^2} - c^2} = c\sqrt{\frac{1}{q^2} - 1} = c\sqrt{\frac{1}{1-\delta} - 1} = c\sqrt{\frac{\delta}{1-\delta}}. \tag{14}$$

Hence

$$b = \frac{\sqrt{1-\delta}}{2} \|\lambda_1 - \lambda_2\| \sqrt{\frac{\delta}{1-\delta}} = \frac{\sqrt{\delta}}{2} \|\lambda_1 - \lambda_2\| . \tag{15}$$

On the other hand we have

$$\nu_q(U) + b = \left\| \frac{q\lambda_1 + q\lambda_2}{2} \right\| = \frac{q}{2} \|\lambda_1 + \lambda_2\| = \frac{\sqrt{1-\delta}}{2} \|\lambda_1 + \lambda_2\| \tag{16}$$

and therefore

$$\begin{aligned}\nu_q(U) &= \frac{\sqrt{1-\delta}}{2}\|\lambda_1+\lambda_2\| - \frac{\sqrt{\delta}}{2}\|\lambda_1-\lambda_2\| \\ &= \frac{1}{2}\left(\sqrt{1-\delta}\|\lambda_1+\lambda_2\| - \sqrt{\delta}\|\lambda_1-\lambda_2\|\right).\end{aligned} \tag{17}$$

Now we need to show that the above expression for the distance $\nu_q(U)$ is valid also for higher dimensions. The boundary of q-numerical ranges for larger matrices is described in [2]. It consists of parts of a few ellipses obtained is an analogous way. Let λ_1 and λ_d be the pair of the most distant eigenvalues of U. Let λ_i and λ_j bo some pair of eigenvalues such that $i, j \neq 1, d$. Let $\widetilde{\nu}_q(U)$ be the distance from zero the ellipse built on λ_i and λ_j in the same way as above. Our goal is to prove that $\widetilde{\nu}_q(U) > \nu_q(U)$.

We note that $\|\lambda_1-\lambda_2\| > \|\lambda_i-\lambda_j\|$. Hence to prove that $\widetilde{\nu}_q(U) > \nu_q(U)$ it suffices to show that $\|\lambda_1+\lambda_2\| < \|\lambda_i+\lambda_j\|$. As all the eigenvalues lie on the unit circle, the from the parallelogram law we have $\|\lambda_1+\lambda_2\|^2 = 4 - \|\lambda_1-\lambda_2\|^2$. Therefore

$$\begin{aligned}\|\lambda_1+\lambda_2\| &= \sqrt{4-\|\lambda_1-\lambda_2\|^2} < \sqrt{4-\|\lambda_i-\lambda_j\|^2} \\ &= \sqrt{4-\left(4-\|\lambda_i+\lambda_j\|^2\right)} = \|\lambda_i+\lambda_j\|.\end{aligned} \tag{18}$$

and thus $\widetilde{\nu}_q(U) > \nu_q(U)$, from which it follows that

$$\nu_{\sqrt{1-\delta}}(U) = \frac{1}{2}\left(\sqrt{1-\delta}\|\lambda_1+\lambda_d\| - \sqrt{\delta}\|\lambda_1-\lambda_d\|\right) \tag{19}$$

holds for any dimension d. The above formula can be easily translated into trigonometric functions where Θ is the angle between λ_1 and λ_d. Hence, we have

$$\nu_{\sqrt{1-\delta}}(U) = \sqrt{1-\delta}\cos\left(\frac{\Theta}{2}\right) - \sqrt{\delta}\sin\left(\frac{\Theta}{2}\right). \tag{20}$$

Therefore,

$$p_{\text{II}} = \nu^2_{\sqrt{1-\delta}}(U\otimes \mathbb{1}) = \nu^2_{\sqrt{1-\delta}}(U) = \left(\sqrt{1-\delta}\cos\left(\frac{\Theta}{2}\right) - \sqrt{\delta}\sin\left(\frac{\Theta}{2}\right)\right)^2. \tag{21}$$

Appendix C. Certification of von Neumann measurements

In this appendix we recall a few technical lemmas necessary to prove the main theorem in the paper. The first lemma is the data processing inequality. This inequality, along with its proof, can be found eg. in [3]. However, to keep this work self-consistent we present our modified version of them.

Lemma 1. *(Data processing inequality) Let $\delta > 0$ and Ω be a positive semidefinite operator such that $\Omega \leq \mathbb{1}$. For any quantum channel Φ and quantum states ρ, σ the following holds*

$$\min_{\Omega:\operatorname{tr}(\Omega\rho)\geq 1-\delta} \operatorname{tr}(\Omega\sigma) \leq \min_{\Omega:\operatorname{tr}(\Omega\Phi(\rho))\geq 1-\delta} \operatorname{tr}(\Omega\Phi(\sigma)). \tag{22}$$

Proof. Let us consider two-point certification of two quantum states ρ and σ with statistical significance δ. To calculate the probability of the type II error, p_{II}, we formulate the problem as

$$\min_{\Omega:\, \mathrm{tr}(\Omega\rho)\geq 1-\delta} \mathrm{tr}(\Omega\sigma). \tag{23}$$

Now, consider the scenario in which we use as processing the quantum channel Φ on states ρ and σ. We want to calculate

$$\min_{\Omega:\mathrm{tr}(\Omega\Phi(\rho))\geq 1-\delta} \mathrm{tr}(\Omega\Phi(\sigma)) \tag{24}$$

which is equivalent to

$$\min_{\Omega:\mathrm{tr}(\Phi^\dagger(\Omega)\rho)\geq 1-\delta} \mathrm{tr}(\Phi^\dagger(\Omega)\sigma). \tag{25}$$

It easy to see that $\Phi^\dagger(\Omega)$ is also a measurement and

$$\{\Phi^\dagger(\Omega) : \mathrm{tr}(\Phi^\dagger(\Omega)\rho) \geq 1-\delta\} \subseteq \{\Omega : \mathrm{tr}(\Omega\rho) \geq 1-\delta\}. \tag{26}$$

Eventually, we obtain the data processing inequality given by

$$\min_{\Omega:\mathrm{tr}(\Omega\rho)\geq 1-\delta} \mathrm{tr}(\Omega\sigma) \leq \min_{\Omega:\mathrm{tr}(\Omega\Phi(\rho))\geq 1-\delta} \mathrm{tr}(\Omega\Phi(\sigma)). \tag{27}$$

□

The following lemma is proved in the work [4].

Lemma 2. *(Lemma 5 from [4], direct implication) Assume that $E_0 \in \mathcal{DU}_d$ satisfies the condition*

$$||\Phi_{UE_0} - \Phi_{\mathbb{1}}||_\diamond = ||\mathcal{P}_U - \mathcal{P}_{\mathbb{1}}||_\diamond < 2. \tag{28}$$

Let λ_1, λ_d be a pair of the most distant eigenvalues of UE_0 and Π_1, Π_d be the projectors onto the subspaces spanned by the eigenvectors corresponding to λ_1 and λ_d, respectively. Then, there exist states ρ_1, ρ_d, satisfying the following conditions

$$\begin{aligned} \rho_1 &= \Pi_1\rho_1\Pi_1 \\ \rho_d &= \Pi_d\rho_d\Pi_d \\ \mathrm{diag}(\rho_1) &= \mathrm{diag}(\rho_d). \end{aligned} \tag{29}$$

The next proposition follows directly from Lemma 2.

Corollary 1. *Let $\rho_0 = \frac{1}{2}\rho_1 + \frac{1}{2}\rho_d$ be the state satisfying conditions given by Eq. (29). Then, for each $i \in \{1, \ldots, d\}$ we have*

$$\mathrm{tr}\left(\sqrt{\rho_0}|i\rangle\langle i|\sqrt{\rho_0}\right) = \mathrm{tr}\left(\sqrt{\rho_0}U|i\rangle\langle i|U^\dagger\sqrt{\rho_0}\right). \tag{30}$$

Moreover, for each $i \in \{1, \ldots, d\}$ such that $\langle i|\rho_0|i\rangle \neq 0$ we get

$$\left|\frac{\langle i|\rho_0 U|i\rangle}{\langle i|\rho_0|i\rangle}\right| = \left|\frac{\lambda_1 + \lambda_d}{2}\right|. \tag{31}$$

Proof. Let $U = \sum_{i=1}^d \lambda_i \Pi_i$, where $\{\Pi_i\}_{i=1}^d$ is a set of orthogonal projectors. Then

$$\begin{aligned}
&\operatorname{tr}\left(\sqrt{\rho_0} U |i\rangle\langle i| U^\dagger \sqrt{\rho_0}\right) = \langle i|U^\dagger \rho U|i\rangle = \langle i|U^\dagger \left(\frac{1}{2}\rho_1 + \frac{1}{2}\rho_d\right) U|i\rangle \\
&= \langle i|U^\dagger \left(\frac{1}{2}\Pi_1\rho_1\Pi_1 + \frac{1}{2}\Pi_d\rho_d\Pi_d\right) U|i\rangle \\
&= \langle i| \left(\sum_{i=1}^d \overline{\lambda_i}\Pi_i^\dagger\right) \left(\frac{1}{2}\Pi_1\rho_1\Pi_1 + \frac{1}{2}\Pi_d\rho_d\Pi_d\right) \left(\sum_{i=1}^d \lambda_i\Pi_i\right) |i\rangle \\
&= \langle i| \left(\frac{1}{2}\rho_1 + \frac{1}{2}\rho_d\right) |i\rangle = \operatorname{tr}\left(\sqrt{\rho_0}|i\rangle\langle i|\sqrt{\rho_0}\right).
\end{aligned} \tag{32}$$

where the third equality follows from Lemma 2.

To prove the second part of the proposition we calculate

$$\begin{aligned}
\left|\frac{\langle i|\rho_0 U|i\rangle}{\langle i|\rho_0|i\rangle}\right| &= \left|\frac{\langle i| \left(\frac{1}{2}\rho_1 + \frac{1}{2}\rho_d\right)\left(\sum_{i=1}^d \lambda_i\Pi_i\right)|i\rangle}{\langle i|\rho_0|i\rangle}\right| \\
&= \left|\frac{\langle i| \sum_{i=1}^d \lambda_i \left(\frac{1}{2}\Pi_1\rho_1\Pi_1 + \frac{1}{2}\Pi_d\rho_d\Pi_d\right)\Pi_i|i\rangle}{\langle i|\rho_0|i\rangle}\right| \\
&= \left|\frac{\langle i| \left(\frac{1}{2}\lambda_1\Pi_1\rho_1\Pi_1 + \frac{1}{2}\lambda_d\Pi_d\rho_d\Pi_d\right)|i\rangle}{\langle i|\rho_0|i\rangle}\right| \\
&= \left|\frac{\langle i| \left(\frac{1}{2}\lambda_1\rho_1 + \frac{1}{2}\lambda_d\rho_d\right)|i\rangle}{\langle i|\rho_0|i\rangle}\right| = \left|\frac{\lambda_1 + \lambda_d}{2}\right|.
\end{aligned} \tag{33}$$

□

Appendix D. Animation of q-numerical range

For an animation of the behavior of the q-numerical range of a unitary matrix $U \in \mathcal{U}_3$ see the attached `gif` file.

Figure 2. An animation of q-numerical range of unitary matrix $U \in \mathcal{U}_3$ with eigenvalues $1, \mathrm{e}^{\frac{\pi \mathrm{i}}{3}}$ and $\mathrm{e}^{\frac{2\pi \mathrm{i}}{3}}$ for all parameters $q \in [0, 1]$.

References

[1] R. Duan, Y. Feng, and M. Ying, “Perfect distinguishability of quantum operations,” *Physical Review Letters*, vol. 103, no. 21, p. 210501, 2009.

[2] C.-K. Li and H. Nakazato, “Some results on the q-numerical,” *Linear and Multilinear Algebra*, vol. 43, no. 4, pp. 385–409, 1998.

[3] L. Wang and R. Renner, “One-shot classical-quantum capacity and hypothesis testing,” *Physical Review Letters*, vol. 108, no. 20, p. 200501, 2012.

[4] Z. Puchała, Ł. Pawela, A. Krawiec, and R. Kukulski, “Strategies for optimal single-shot discrimination of quantum measurements,” *Physical Review A*, vol. 98, no. 4, p. 042103, 2018.
