# Supplementary figures and images for: On the optimal certification of von Neumann measurements
Source: Sci Rep. 2021 Feb 11;11:3623. doi: 10.1038/s41598-021-81325-1 (PMC7878518; doi:10.1038/s41598-021-81325-1)

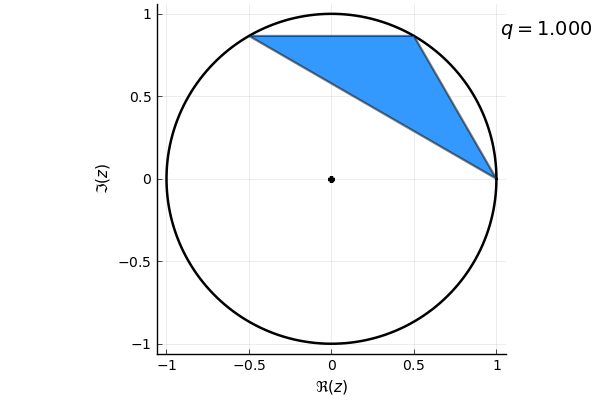

Supplement: Supplementary file 2 — Supplementary Information 2. [file 41598_2021_81325_MOESM2_ESM.gif]
